# Exome sequences and multi‐environment field trials elucidate the genetic basis of adaptation in barley

**DOI:** 10.1111/tpj.14414

**Published:** 2019-06-27

**Authors:** Daniela Bustos‐Korts, Ian K. Dawson, Joanne Russell, Alessandro Tondelli, Davide Guerra, Chiara Ferrandi, Francesco Strozzi, Ezequiel L. Nicolazzi, Marta Molnar‐Lang, Hakan Ozkan, Maria Megyeri, Peter Miko, Esra Çakır, Enes Yakışır, Noemi Trabanco, Stefano Delbono, Stylianos Kyriakidis, Allan Booth, Davide Cammarano, Martin Mascher, Peter Werner, Luigi Cattivelli, Laura Rossini, Nils Stein, Benjamin Kilian, Robbie Waugh, Fred A. van Eeuwijk

**Affiliations:** ^1^ Biometris Wageningen University and Research Centre PO Box 16 6700 AC Wageningen The Netherlands; ^2^ Cell and Molecular Sciences James Hutton Institute Invergowrie, Dundee UK; ^3^ CREA – Research Centre for Genomics and Bioinformatics Via S. Protaso 302 29017 Fiorenzuola d'Arda Italy; ^4^ PTP Science Park Via Einstein, Loc. Cascina Codazza 26900 Lodi Italy; ^5^ Agricultural Institute Centre for Agricultural Research Hungarian Academy of Sciences 2462 Martonvásár Hungary; ^6^ University of Çukurova Faculty of Agriculture Department of Field Crops 01330 Adana Turkey; ^7^ Bahri Dagdas International Agricultural Research Institute Konya Turkey; ^8^ Università degli Studi di Milano – DiSAA Via Celoria 2 20133 Milano Italy; ^9^ Leibniz Institute of Plant Genetics and Crop Plant Research (IPK) 06466 Seeland Germany; ^10^ KWS UK Ltd 56 Church Street Thriplow, Royston SG8 7RE UK; ^11^ Division of Plant Sciences School of Life Sciences University of Dundee Dow Street Dundee DD1 5EH UK; ^12^ Present address: Global Crop Diversity Trust Platz der Vereinten Nationen 7 53113 Bonn Germany

**Keywords:** barley, common garden trials, exome sequence haplotypes, genetic diversity, genotype‐by‐environment interactions, adaptation, *H. vulgare* ssp. *vulgare*

## Abstract

Broadening the genetic base of crops is crucial for developing varieties to respond to global agricultural challenges such as climate change. Here, we analysed a diverse panel of 371 domesticated lines of the model crop barley to explore the genetics of crop adaptation. We first collected exome sequence data and phenotypes of key life history traits from contrasting multi‐environment common garden trials. Then we applied refined statistical methods, including some based on exomic haplotype states, for genotype‐by‐environment (G×E) modelling. Sub‐populations defined from exomic profiles were coincident with barley's biology, geography and history, and explained a high proportion of trial phenotypic variance. Clear G×E interactions indicated adaptation profiles that varied for landraces and cultivars. Exploration of circadian clock‐related genes, associated with the environmentally adaptive days to heading trait (crucial for the crop's spread from the Fertile Crescent), illustrated complexities in G×E effect directions, and the importance of latitudinally based genic context in the expression of large‐effect alleles. Our analysis supports a gene‐level scientific understanding of crop adaption and leads to practical opportunities for crop improvement, allowing the prioritisation of genomic regions and particular sets of lines for breeding efforts seeking to cope with climate change and other stresses.

## Significance Statement

We analysed exome sequence data and life history traits measured in multi‐environment trials of a diverse panel of 371 domesticated barley lines to explore the genetics of crop adaptation. G×E interactions indicated adaptation profiles that varied for sub‐populations defined from exomic data. Exploration of circadian clock‐related genes associated with days to heading revealed complexities in G×E effect directions, and the importance of latitudinally based genic context in the expression of large effect alleles. Analysis provides practical information for future crop breeding.

## Introduction

Barley (*Hordeum vulgare* ssp. *vulgare*) is globally the fourth most important cereal crop after maize, rice and wheat (http://faostat.fao.org). Its cultivation across a wide range of environments makes it a relevant crop for exploring farmers’ adaption strategies to anthropogenic climate change (Dawson *et al*., 2015; Khoury and Achicanoy, 2016), while its founder status in the development of agriculture in the Fertile Crescent 10 millennia ago has made it a focus of crop evolutionary studies (Russell *et al*., 2016). In particular, the expansion in latitude and longitude of the crop to production areas outside the Fertile Crescent means that it is regarded as a model for understanding crop adaptation during anthropogenic range extension. With its structurally simpler genome (2*n* = 2*x* = 14) than bread wheat (*Triticum aestivum*) (2*n* = 6*x* = 42) (Brenchley *et al*., 2012; Mayer *et al*., 2012), barley can be considered a model for the latter crop that originated in the same geographical region and has also spread widely to become one of the world's most significant food sources.

Traits of key importance for responding to a wide range of biotic and abiotic stresses have been identified, characterized and reported for barley (Dawson *et al*., 2015). Flowering time, reflected in the days to heading (DTH) trait, which is the time elapsed between planting of the crop and ear emergence, is an important adaptive feature in the spread of many cereal crops from their origins, enabling the matching of reproductive seed production to appropriate environmental conditions of temperature, precipitation, evapotranspiration, light and other variables (Nakamichi, 2015). Variation in DTH has underpinned barley's spread from the Fertile Crescent to more extreme latitudes (Comadran *et al*., 2012), and circadian clock‐related genes that help control the trait in the crop have previously been described (Calixto *et al*., 2015). Understanding genetic variation in DTH is therefore of academic interest for exploring the domestication and expansion of cereal crops and of practical importance for addressing the future climate change‐related shifts that will be required in production. Possible climate‐related responses may be complex and at first sight appear counter‐intuitive: for example, avoiding summer droughts caused by climate warming could in the case of barley be achieved by making varieties more cold tolerant so that they can be planted in the autumn rather than the spring, with the crop then flowering and maturing earlier the following season (Fisk *et al*., 2013). The spring and winter growth habits of different barley varieties, with their different responses to cold, heat and light, thus make the crop particularly interesting for exploring environmentally adaptive responses in agriculture (Dawson *et al*., 2015).

Although understanding the underlying genetics of adaptation to varying environments and broadening the genetic base of crops for flowering, flowering‐associated and other important traits are considered important for developing varieties adapted to future production conditions (Ellis *et al*., 2000; Tester and Langridge, 2010), the genetic diversity deployed in breeding programmes has to date been limited due to modern varietal selection processes (Kilian *et al*., 2006). Broader genetic diversity in landraces and wild progenitors has, however, been maintained in genebanks worldwide for many crops, including barley (Igartua *et al*., 1998; Knüpffer, 2009; Muñoz‐Amatriaín *et al*., 2014). This diversity is now being made more accessible to breeders through the adoption of advanced methods that are better able to discover, dissect out and employ relevant variation (Dempewolf *et al*., 2017; Mascher *et al*., 2017). A crucial enabling factor is comprehensive genotyping and phenotyping of germplasm across relevant contrasting environments to understand genomic drivers of adaptation (Muñoz‐Amatriaín *et al*., 2014). In terms of genotyping, exome sequencing has become an established approach in recent years. This method focuses only on the gene space of organisms and thereby reduces the costs of sequencing and analysis compared with whole‐genome approaches, making it highly appropriate for crops such as barley that have a very high proportion of non‐genic genomic DNA (Mascher *et al*., 2017). The exome approach for barley was initially described by Mascher *et al*. (2013) and first applied seriously to examine the crop's adaptive responses in an analysis of 267 landrace and wild relative (*H. vulgare* ssp. *spontaneum*, wild barley) lines by Russell *et al*. (2016). Their analysis incorporated a combination of exome capture sequencing, field trials, bioclimatic data and various statistical approaches to landscape genomics, to initially explore drivers of environmental adaption.

Here, we report a combined genomic and phenomic analysis of 371 domesticated barley lines that allows for a more detailed study of adaptive features in the crop. The tested germplasm was carefully chosen through wide consultation with breeders, genebank curators and researchers. Lines were, in parallel, exome captured and field trialled, with common garden experiments taking place across a range of environments (locations and seasons), and phenotyped for a number of fundamental traits, including DTH, 1000‐grain weight, plant height and awn length. We then applied refined statistical methods to jointly model detailed exome capture and multi‐environment phenotypic data sets in an analysis of variation. We provide evidence extending the scientific understanding of crop adaption that leads to practical opportunities for crop improvement. We also illustrate how environmental adaption can be further dissected through an in‐depth examination of the control of DTH, which involved exploring geographical patterns of variation of key flowering‐related gene haplotype states and phenotypes.

## Results

### Exome sequences reveal substantial variation in the barley collection

Exome sequences that revealed substantial variation in the barley gene pool were obtained and validated. Data were successfully derived for 403 genotypes from the WHEALBI research project (EU FP7 no. FP7‐613556), comprising formally bred cultivars (henceforth referred to simply as cultivars), landraces and wild barley lines (Table S1 in the online Supporting Information). This source material represents a range of worldwide barley genetic diversity, assembled to quantify variation and explore adaptation, to determine possible breeding responses to environmental change (http://www.whealbi.eu/). More than 64 million single nucleotide polymorphisms (SNPs) were extracted from sequences, which, after applying a quality criterion of at least 80% of genotypes being represented at a SNP locus, was reduced to just under 2.1 million SNPs (Table S2). All seven of barley's chromosomes (1H to 7H) were well covered (Figure S1), with a range of 262 014 (6H) to 334 501 (2H) polymorphisms and a median distance between SNPs of between 14 and 27 bp, although a few large gaps in coverage remained, especially close to centromeres. From this initial data set, we used information for 371 domesticated barleys, consisting of cultivars and landraces of both two‐ and six‐rowed types, for further analysis. Of the excluded genotypes from the initial total of 403, 22 were wild barley and another 10 did not pass phenotypic or other, genotypic, quality criteria. Considering only the 371 genotypes, and after we applied a minor allele frequency (MAF) filter of ≥0.05, 435 431 SNPs remained for analysis. (See Experimental procedures for more information on genotype exclusion and our choice of MAF.)

### Population structure in barley corresponds with row type, geographical origin, breeding history and growth habit

**Figure 1:**
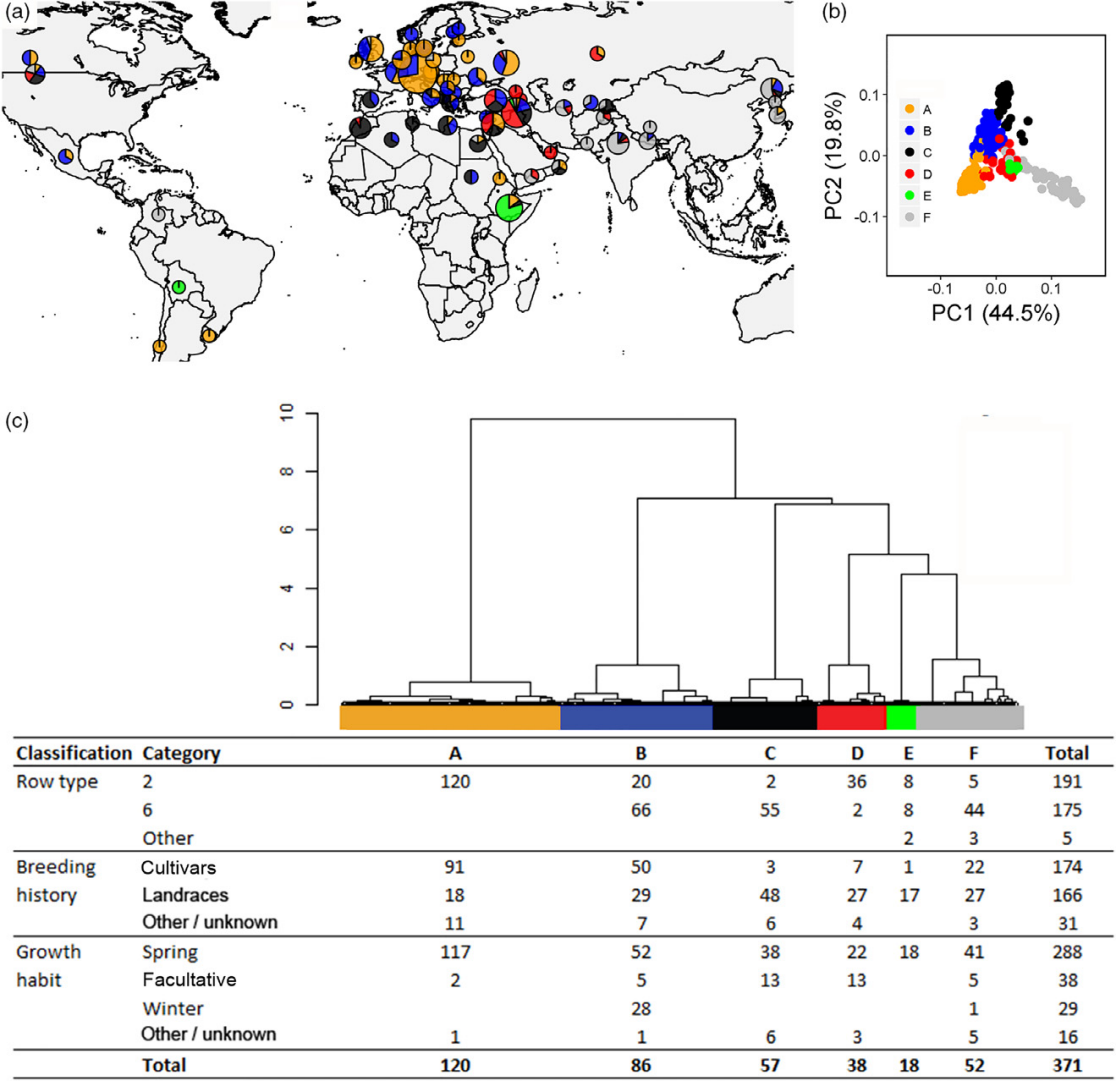
Geographical composition and passport data of the barley panel. (a) Geographical distribution of six sub‐populations of genotypes identified through cluster analysis of significant kinship principal components for 371 barley genotypes. Pie chart size reflects the number of genotypes collected from a particular geographical region, while slice size shows the proportion of genotypes belonging to a particular sub‐population. (b) Principal components biplot of the kinship matrix, showing the classification of genotypes into the six sub‐populations. (c) Hierarchical clustering based on Ward distances, and composition of each of the six sub‐populations, considering row type, breeding history and growth habit classifications. Group A was composed of two‐rowed types, most being European cultivars of spring habit. Group B was primarily six‐rowed types, although a substantial subset was two‐rowed. Most genotypes in this sub‐population were European cultivars, but a sizeable subset was landrace material. Sub‐population B contained all except one of the winter barley lines included in the study (the other winter habit accession grouped to sub‐population F). Sub‐population C consisted primarily of six‐rowed landraces of spring growth habit that came from the Mediterranean area, with some spring facultative habit (cold tolerant, vernalisation unresponsive; Von Zitzewitz *et al*., 2005) lines. Sub‐population D consisted primarily of two‐rowed landraces exhibiting spring or facultative growth habit from the Fertile Crescent. Sub‐population E was a small group of 18 genotypes of spring habit that were primarily Ethiopian landraces, with a mixture of two‐ and six‐rowed types, plus two *intermedium* lines (an intermediate state between the standard two‐ and six‐rowed forms, characterized by enlarged, partially male fertile, lateral spikelets; Ramsay *et al*., 2011). Sub‐population F was composed mostly of six‐rowed spring habit lines from Asia and the Middle East.

Analysis of 371 landrace and cultivar exome profiles revealed significant genetic structuring. The method that we applied, in which genomic population structure was characterized taking linkage disequilibrium (LD) into account when sampling SNPs to construct a kinship matrix (see Experimental procedures and Table S3), identified six barley sub‐populations (A to F) that approximately coincided with row type, geographical origins, breeding histories and growth habit classifications (Figure 1).

### Landscape genomics indicate significant regional geographical structuring

We identified a well‐defined subset of spring growth habit barleys (*n *=* *174, comprising 111 cultivars and 63 landraces) from our initial set of 371 domesticated accessions. Because this subset of accessions excludes winter growth habit lines, the confounding factor of vernalisation in determining DTH has been removed. The genotypes chosen in our subset were also all non‐tropical, such that day length adaptation at specific sampling latitudes should be an important feature of the crop. We later use these lines to analyse the geographical details of control of DTH by specific gene sequences, as presented in subsequent sections. We first subjected this subset of 174 accessions to a landscape genomics analysis to determine the overall pattern of geospatially related genetic structure in the spring habit crop. Spatial principal component analysis (sPCA) revealed significant overall geographically based genetic structuring. We found that for summed genomic data a ‘regional’ structure of allelic frequencies predominated over a local one (Figure S2a), with these frequencies showing large spatial autocorrelation (Figure S2b). The relevance of regional over local structure indicated geographical gradients in the overall distribution of genetic variation. The main gradient, as represented by sPCA1 and sPCA2, was from north‐western Europe towards southern latitudes. Northern European genotypes were clearly different from those surrounding the Mediterranean, while a differentiated group of accessions was also located in the Middle East (Figure S2c). This pattern of genetic variation corresponds with our earlier geographical analysis of all 371 accessions (Figure 1a). As we report later, it also corresponds with differentiation at specific flowering‐related genes for our larger sample set (Figure S12). These results further indicate the presence of regional geographical structuring that extends to equatorial regions, with Ethiopian landrace barley differentiated from other sample locations.

### Chromosome‐level analysis reveals strong localised differentiation by barley category and highly variable linkage disequilibrium decay

Further analysis of row type and breeding history as drivers of genetic differentiation (see Experimental procedures) in our panel of 371 domesticated barley lines revealed strong differentiation at specific chromosome positions. For two‐rowed versus six‐rowed lines (Figure S3a), high differentiation was observed at relatively distal portions of chromosome 2H (largest *F*_st_ = 0.82 at 648.3 Mbp) and 4H (largest *F*_st_ = 0.94 at 17.4 Mbp), close to the locations of the known row‐type genes *Vrs1* (652.1 Mbp on 2H; Komatsuda *et al*., 2007) and *INT‐C* (17.6 Mbp on 4H; Ramsay *et al*., 2011), respectively. There was also relatively high genetic differentiation at a position on chromosome 5H not coincident with any known gene regulating row type, indicating a candidate region for further exploration. Considering differentiation between cultivars and landraces, genomic regions with clear contrasts were also evident (Figure S3b), especially close to known genes for row type and flowering time. This may, however, reflect the specific structure of our germplasm panel where most cultivars are two‐rowed (including European malting barleys) and most landraces are six‐rowed (in particular, the large group of Mediterranean accessions), so it is not straightforward to draw firm conclusions based on breeding history per se from the comparison.

**Figure 2:**
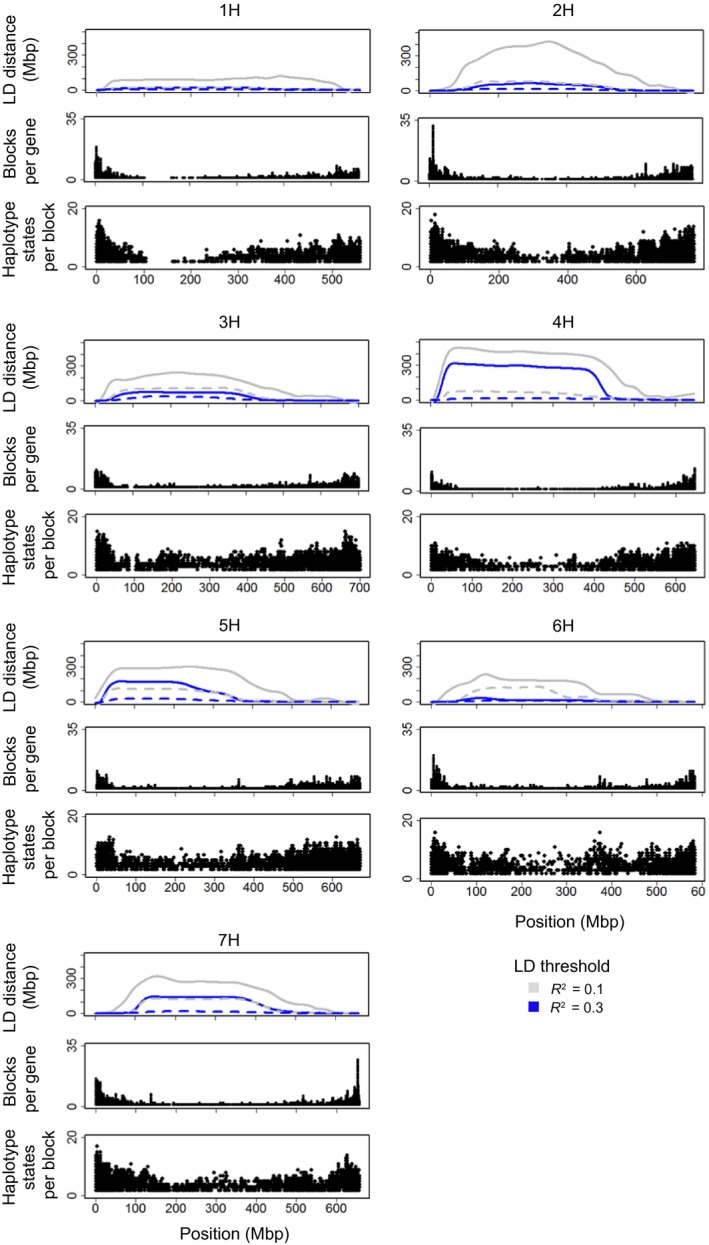
Linkage disequilibrium (LD) and haplotype profiles for barley chromosomes for 371 domesticated barley genotypes.The top of each chromosome's schematic shows LD decay profiles by indicating the genetic distance for LD to fall below a given threshold with and without correction for population structure, using dotted or continuous (grey and blue) lines, respectively. The middle section of each chromosome's sub‐figure shows the number of LD (subsequently defined as haplotype) blocks per gene identified with the method of Gabriel *et al*. (2002). The bottom section of each sub‐figure shows the number of haplotype states per block. The *x*‐axis represents physical distances.

As expected in barley (Cockram *et al*., 2010), LD profiles along chromosomes characterized using the sliding windows method (see Experimental procedures) indicated large non‐recombinant centromeric and peri‐centromeric regions, with variable decay patterns (Figure 2). Overall, 2H and 4H had higher LD than other chromosomes. The LD pattern is largely expected to be driven by sub‐population structure (see Experimental procedures), with the profiles for 2H and 4H possibly shaped by sub‐population specificities around loci regulating flowering time [the circadian clock photoperiod response gene *HvPPD‐H1* (Turner *et al*., 2005) and the earliness per se gene *HvCEN* (Comadran *et al*., 2012), both on 2H] and row type (*Vrs1* on 2H and *INT‐C* on 4H). Regardless its basis, large observed differences in LD indicate the importance of methods for achieving local and flexible characterization, as related further in Experimental procedures.

Single nucleotide polymorphisms in high LD that can be grouped to form haplotype blocks (Daly *et al*., 2001) indicated that genes in distal chromosome regions had larger numbers of blocks, with each block on average having more haplotype states (Figure 2). While individual genes normally comprised between one and five blocks, 32 were observed for *HORVU2Hr1G004930* [a zinc finger (Ran‐binding) family protein on 2H at 11.1 Mbp] and 27 for *HORVU7Hr1G121250* (an aquaporin‐like superfamily protein on 7H at 653.5 Mbp). Generally, individual haplotype blocks had fewer than 10 haplotype states, although 18 were observed for *HORVU2Hr1G005650* (a cullin‐associated NEDD8‐dissociated protein 1 on 2H at 12.2 Mbp).

### G×E modelling of field trial data reveals diverse adaptation patterns in barley

Days to heading, plant height, 1000‐grain weight and awn length phenotypes evaluated in three to five widely different environments (Tables S4 and S5) for our panel of 371 domesticated barley lines are given in Table S1 and summarised in Figure S4. Overall, all traits and environments had a large heritability (Table S5), showing that the phenotypic data were useful for further genetic analyses. G×E analysis indicated that the panel exhibited diverse adaptation with extensive phenotypic plasticity. Considering the season of sowing, as expected barley clearly headed earlier (with reference to days from planting date) in spring‐planted than winter‐planted trials (by around 79 days; Figure S4), indicating the importance of phenotypic plasticity in DTH in promoting flowering under favourable environments. Spring‐ and winter‐sown trials, however, showed similar population means for our other analysed traits of grain weight, plant height and awn length.

**Figure 3:**
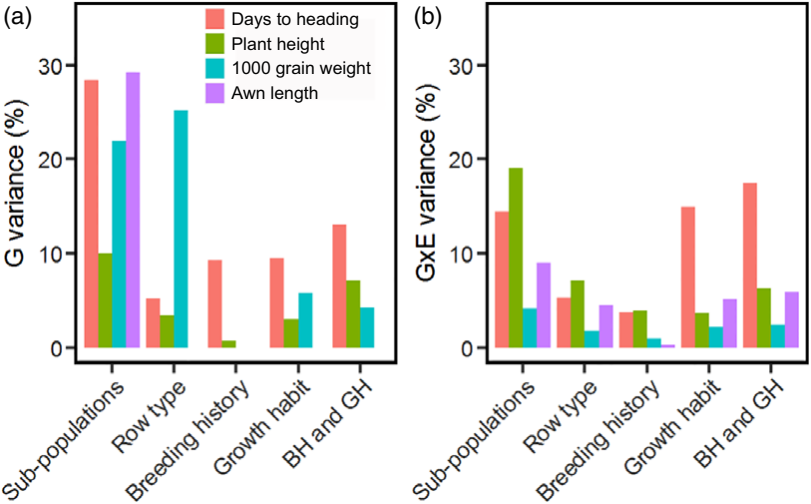
Phenotypic variance explained by categories of barley genotypes.(a) Genotype main effect.(b) Genotype by environment (G×E) effect across multi‐environment field trials.In each case, the percentage variance is shown for five barley categories: sub‐populations identified from exome data (six sub‐populations), row type (two‐row or six‐row), breeding history (cultivar or landrace), growth habit (spring, spring facultative or winter), and the combination of breeding history and growth habit, indicated as ‘BH and GH’ (spring cultivar, winter cultivar, spring landrace, winter landrace, spring facultative landrace). Some of the total set of 371 barley accessions were excluded from analysis because they were unclassified by a specific sub‐category (see Figure 1c). Missing bars indicate that the percentage variance explained was equal to zero. As expected from earlier analysis of accession partitioning (Figure 1), sub‐populations are effective in describing variation. Other interesting features are discussed in the text.

G×E amounted to between 50% and 81% of the variance for the genotypic main effect for measured traits (Table S6), indicating that our barley panel was diverse in its mechanisms of adaptation to environment. As expected based on genomic differentiation, classifications by sub‐population, row type, breeding history and growth habit differed in the amount of genotypic (Figure 3a) and G×E variance (Figure 3b) described. Our six defined sub‐populations generally explained the largest amount of G×E variance for genotypes across traits [14% for DTH (but see also effect of growth habit), 18% for plant height, 4% for grain weight and 8% for awn length], thus emphasising the value of explicit genotype characterizations for explaining adaptive responses.

**Figure 4:**
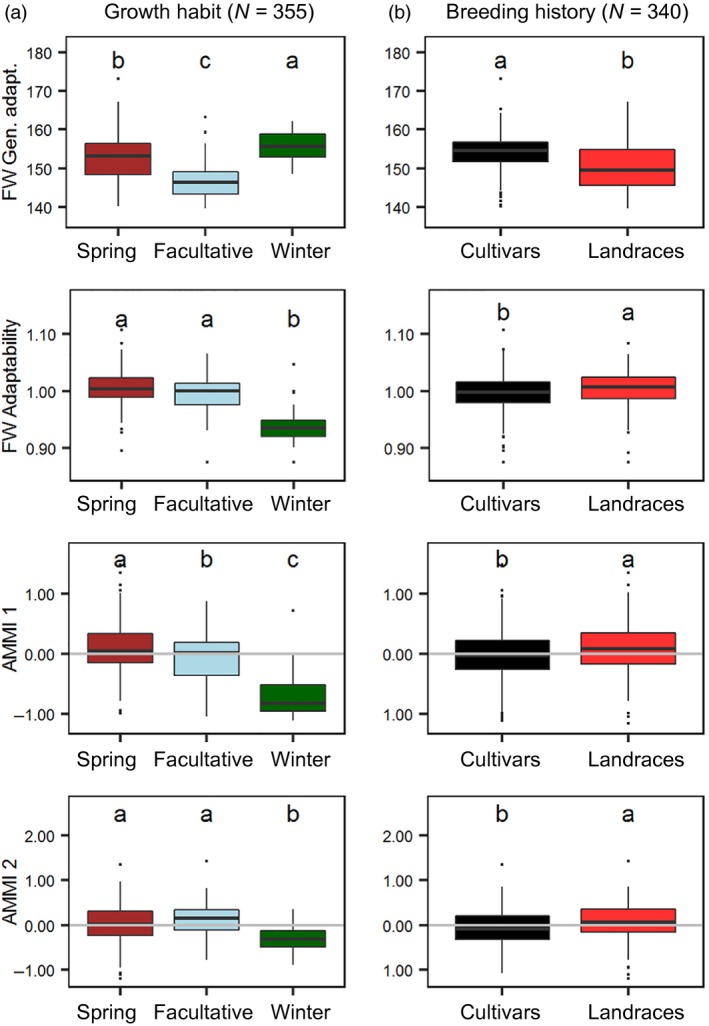
Box plots of general adaptation and adaptability, as characterized by Finlay–Wilkinson regression and an Additive Main effect and Multiplicative Interaction (AMMI) model fitted to days to heading (DTH) across multi‐environment trials.Barley genotypes were categorised by (a) growth habit (spring facultative, *n = *38; spring, *n = *288; winter, *n = *29) and (b) breeding history (cultivar, *n = *174; landrace, *n = *166). In the top row of panels of (a) and (b), Finlay–Wilkinson general adaptation, corresponding to mean performance across environments, is shown. In the second row of panels of (a) and (b), Finlay–Wilkinson adaptability, relating genotype sensitivity to the environmental gradient, is indicated. Here, genotypes that have a value (for the slope) of greater and less than one demonstrate more and less sensitivity to the environment compared with the population mean, respectively. In the third and final rows of panels of (a) and (b), DTH is explored further by AMMI, with scores for AMMI 1 and AMMI 2 shown, respectively, by row, to indicate categorised genotype contributions to genotype by environment interaction (G×E) (see also Figure S6). In these plots, genotypes the deviate most from zero (positively or negatively) contribute more to G×E (i.e. genotypes with scores close to zero deviate little from the general population response along the environmental gradient). For growth habit, the AMMI plots indicate the contrast between spring and winter habit types across trial sowing date. Winter genotypes have strongly negative scores, indicating that their DTH is relatively more delayed in spring trials compared with other lines. For breeding history, landraces show more positive scores in AMMI plots, indicating that their DTH is conversely relatively more delayed in winter trials compared with spring trials. Some of our panel of 371 genotypes were excluded from analysis because they were unclassified by specific sub‐categories (see Figure 1c). Letters show significant differences based on a Tukey test (*P* <0.05).

Focusing on G×E variance for DTH, season of sowing was clearly the main driver for the interaction, with the ranking of our identified sub‐populations changing across season (Figure S4). Finlay–Wilkinson regressions that allowed estimation of general adaptation (mean performance across environments) and adaptability (sensitivity to environments, as related further in Experimental procedures) indicated that a spring growth habit and a landrace breeding history determined quicker heading across all environments (top panels of Figure 4a,b). The defining environmental contrast was that between winter‐sown (long DTH) and spring‐sown (short DTH) trials, so that adaptability (second row of panels in Figure 4a,b) reflects the relative lateness of genotypes in winter trials versus earliness in spring trials (see also Figure S5). The same winter‐sown versus spring‐sown contrast determined the first additive main effect and multiplicative interaction (AMMI) axis (AMMI 1), with genotype scores being similar to Finlay–Wilkinson adaptabilities (compare the second and third rows of the panels of Figure 4a,b). Scores for sub‐population B (see Figure 1) genotypes were mostly negative on AMMI 1, and so were the environmental scores for the spring trials (blue vectors in Figure S6), implying that flowering time for this sub‐population was relatively more delayed in spring trials than that for other sub‐populations. Scores on the second AMMI axis (AMMI 2) (bottom panels of Figure 4a,b) corresponded to a contrast between Italian winter‐sown and other winter trials in Scotland and Hungary (Figure S6). Deviation from the origin for genotypes in AMMI biplots is proportional to adaptability, as related further in Experimental procedures, indicating that this was greater for winter habit barleys (Figure 4a). This is consistent with delayed flowering of winter barley lines under spring sowing due to sub‐optimal vernalisation (vernalisation generally accelerates flowering in winter barleys only; Von Zitzewitz *et al*., 2005). In the case of landraces, which tend to have a positive interaction with winter trials (Figures 4b and S5a), many lines (especially from the earliest heading subgroups C and D; Figures 1 and S4) come from the Mediterranean area, where earliness is effective in escaping terminal drought stress. As most landraces have a spring growth habit, their DTH is relatively more delayed in winter‐sown trials than in spring‐sown trials.

Considering G×E for plant height (Figure 3b), this was driven by the positive interaction between sub‐populations B and C (Figure 1) with the environments ‘Hungary winter’ and ‘Italy winter’ (genotypic and environmental scores in similar directions in relation to the origin of a genotype plus G×E (GGE) biplot; see Figure S5b), which may be explained by Mediterranean landraces growing taller under temperate European climates with high thermal time and plenty of rainfall. In the case of grain weight and awn length traits, sub‐populations contributed primarily to the main genotype effect and less to G×E (compare Figure 3a and b), which is reflected in the less clear separation between sub‐population groups in GGE biplots (Figure S5c,d).

### Multi‐environment genome‐wide association scans support the power of barley germplasm panels and multi‐environment field phenotyping for genetic trait analysis

**Figure 5:**
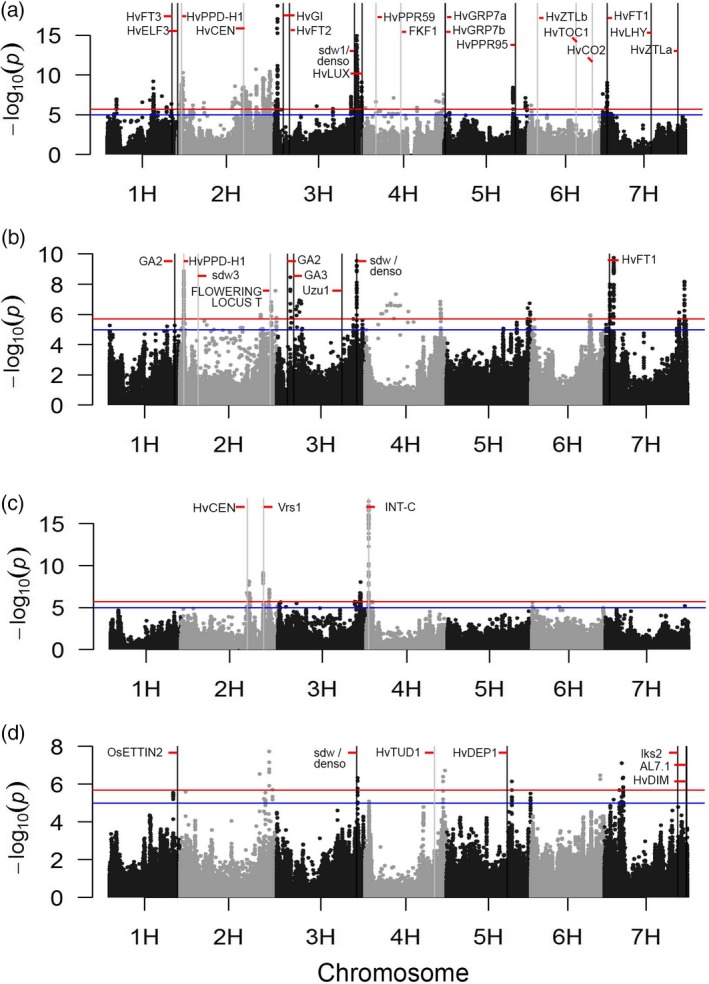
Multi‐environment genome‐wide association scans of individual single nucleotide polymorphism profiles. Manhattan plots for (a) days to heading, (b) plant height, (c) 1000‐grain weight and (d) awn length for 371 domesticated barley lines. Blue and red lines indicate a multiple testing correction of α = 0.05 and α = 0.01, respectively. Vertical lines indicate positions of some important known genes reported in the literature. The x‐axis represents physical distances.

Multi‐environment genome‐wide association scans (GWAS) of individual SNPs (Figure 5) and haplotype states (Figure S7) demonstrated large quantitative trait loci (QTLs) for the four phenotypic traits under investigation, with clear peaks coincident with or proximate to known trait‐related genes, indicating that our genotype panel and common garden trials provide a powerful platform for identifying the genetic basis of barley adaptation across environments.

**Figure 6:**
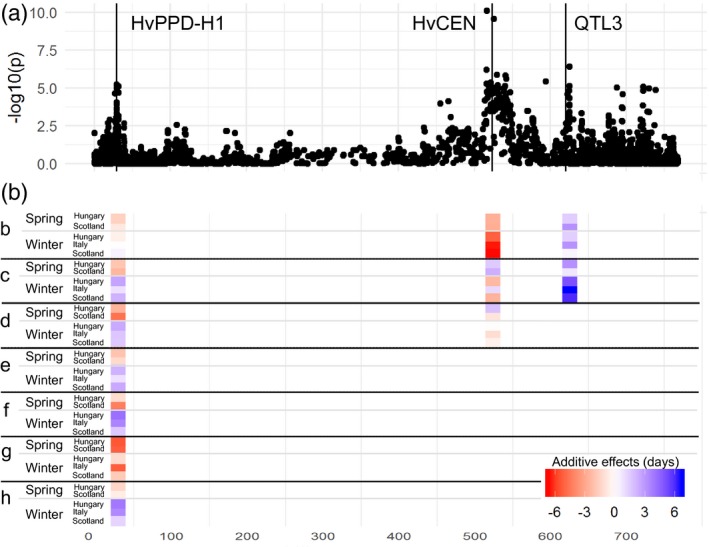
(a) Manhattan plot for days to heading (DTH) for 371 domesticated barley lines and (b) additive effects for specific haplotype states for *HvPPD‐H1*,* HvCEN* and quantitative trait locus (QTL) 3. In the last case, haplotype states are for the haplotype block associated with the identified QTL position itself, which is close to but not coincident with *HvCO4*. In (b), rows indicate the additive substitution effects, in days, of specific haplotype states (all with reference to the most common haplotype state, haplotype *a*, chosen as the baseline; see also Table S8). Haplotypes are shown in descending order of frequency of occurrence (e.g. haplotype *b* is the most common after *a*), meaning that the effects of the haplotype states closer to the top of the schematic are more definitive (haplotype state sample sizes given in Table S8). For *HvPPD‐H1*, haplotype states *a* and *b* both correspond to *photoperiod‐insensitive* alleles and haplotype states *c*,* d*,* e*,* f*,* g* and *h* all to *photoperiod‐sensitive* alleles according to the classification of Turner *et al*. (2005). For *HvCEN*, haplotype states *b* and *d* correspond to *early* alleles and haplotype states *a* and *c* correspond to *late* alleles according to the classification of Comadran *et al*. (2012). The *x*‐axis represents physical distance in Mbp.

The most significant associations between individual SNPs and DTH (Figure 5a) were observed on chromosomes 2H and 3H. On 3H a QTL located at 650.6 Mbp was close to the semi‐dwarfing gene *sdw1*/*denso* (encoding a gibberellin 20‐oxidase 3, 634.1 Mbp; Jia *et al*., 2009). The most significant QTL evident on 2H, positioned at 620.1 Mbp, was relatively close to, although still some distance from, the flowering‐associated gene *HvCEN* (523.4 Mbp). Specifically considering GWAS for chromosome 2H based instead on haplotype states (rather than SNPs; Figure 6a, Table S7), clear peaks were, however, observed with both *HvPPD‐H1* and *HvCEN*, genes that regulate flowering time [causal variation at these circadian clock‐related genes was investigated by Turner *et al*. (2005) and Comadran *et al*. (2012), respectively]. In addition, this haplotype‐based analysis showed a third clear QTL (QTL3) on 2H at 625.4 Mbp close to the position of *HORVU2Hr1G085910* that encodes a zinc finger protein CONSTANS‐LIKE 4 that is a candidate for flowering time regulation (*HvCO4*, 620.6 Mb, Griffiths *et al*., 2003). Additive effects for DTH around the most significant genic haplotypes on 2H coincided with the general G×E pattern, especially for the QTL near *HvPPD‐H1*, with clear differences for the spring‐sown and winter‐sown trials (Figure 6b). For *HvPPD‐H1*, whereas haplotype states *a* and *b* showed similar effects in spring and winter trials, consistent with their classification as *photoperiod‐insensitive* alleles (Turner *et al*., 2005; see also Table S8), the haplotype states of *c*,* d*,* e*,* f* and *h* showed a contrasting response across trial season (accelerating and delaying DTH in spring‐sown and winter‐sown trials, respectively; Figure 6b, Table S8), consistent with their classification as all *photoperiod‐sensitive* alleles (Turner *et al*., 2005). For *HvCEN*, no haplotype effects consistently contrasted across trial sowing season. Haplotype state *b,* however, showed a clear negative effect on DTH (i.e. earlier heading) across all trial environments, agreeing with its classification by Comadran *et al*. (2012) as an *early* allele in contrast to the *late* allele *a* (Figure 6b, Table S8). (The frequencies and SNP allelic compositions of haplotype states at *HvPPD‐H1* and *HvCEN* are given in Table S9, along with those for other clock‐related genes discussed below.) With further reference to DTH and haplotype states on 2H, the haplotype block associated with QTL3 (Figure 6b) had three haplotype conditions, with *b* and *c* leading to delayed flowering compared with *a*, and no obvious QTL×E pattern. Although QTL profiles based on haplotype‐based and single SNP‐based GWAS analyses were generally similar, QTLs were placed closer to known genes in the former case, demonstrating the added value of this approach (Figures 5, 6 and S7). This could relate to the larger number of allelic states revealed when using haplotype blocks that allows more flexibility in G×E modelling.

For plant height, GWAS based on single SNPs (Figure 5b) revealed a large QTL on 3H (at 634.4 Mbp) that also featured in an analysis based on haplotypes (Figure S7b, Table S7) and was very close to the *sdw1*/*denso* gene (634.1 Mbp) known to be involved in determining plant stature (Jia *et al*., 2009). Analysis based on single SNPs and haplotypes also revealed a clear QTL at the beginning of 2H (at 29.2 Mbp) that coincided with *HvPPD‐H1*, as well as a QTL on 7H (at 41.1 Mbp) close to *HvFT1* (39.6 Mbp), a gene known to link vernalisation and photoperiod pathways (Faure *et al*., 2007). This suggests that mechanisms for regulating flowering time also impact plant height, coinciding with Tondelli *et al*. (2014).

As expected, 1000‐grain weight was clearly affected by row‐type genes, with *INT‐C* (17.6 Mbp on 4H) showing as a large‐effect QTL, with both single SNP‐ and haplotype‐based GWAS analyses (Figures 5c and S7c, Table S7). A large QTL for 1000‐grain weight was also located on 2H (at 649.8 Mbp) close to the row gene *Vrs1* (652.1 Mbp). A second QTL on 2H, close to *HvCEN*, suggested that *HvCEN* may have a pleiotropic effect on grain weight.

In the case of awn length, the haplotype‐based GWAS scan showed a clear QTL at the end of chromosome 1H (547 Mbp; Figure S7d, Table S7). Locus *HORVU1Hr1G087460.6* is located close to this position (at 539.4 Mbp). We identified through sequence homology searches that this locus coded for an orthologue of rice AUXIN RESPONSE FACTOR 2 (*OsETTIN2*), a gene highly expressed in developing inflorescences of rice that is essential in awn development, along with the DROOPING LEAF (*DL*) gene (Toriba & Hirano, 2014). While associations with genes known to be involved in determining awn development in barley, such as *HvDEP1* (Wendt *et al*., 2016) and *HvTUD1* (Braumann *et al*., 2018), were not significant in our haplotype‐based analysis, the identification of a QTL close to the *OsETTIN2* orthologue suggests a promising candidate for the control of awn length in the crop. There was also a QTL at 642.6 Mbp on chromosome 3H (observed especially for SNP‐based GWAS) that corresponded to the region harbouring *sdw1*/*denso* (634.1 Mbp), suggesting a pleiotropic effect of the semi‐dwarfing gene (Figure 5d).

### Dissecting adaptation and the genetic control of DTH confirms the importance of known circadian clock‐related genes in barley

The DTH trait underpins barley's spread from the Fertile Crescent to more extreme latitudes. We therefore further analysed haplotypes associated with flowering‐associated, circadian clock‐related genes that determine DTH. The suite of genes we chose for analysis was identified by Calixto *et al*. (2015) and analysed by Russell *et al*. (2016) for adaptive responses in the barley crop and wild barley. We were able to construct haplotypes for 13 of the 19 genes reported by Russell *et al*. (2016). As expected, these genes explained a large proportion of phenotypic variation for DTH (Figure S8). Most important was *HvPPD‐H1*, which explained 33% of the main genotypic effect (Figure S8a) and 12% of the G×E variance (Figure S8b). *HvCEN*, as for *HvPPD‐H1* located on chromosome 2H, also had important effects, explaining 21% of the main genotypic effect and 5% of the G×E variance. The QTL effects were on occasions different between spring‐sown and winter‐sown trials (Table S8); as already noted, this was particularly clear for haplotype states at *HvPPD‐H1*, where additive effects were negative in spring‐sown trials (accelerating flowering) and positive in winter‐sown trials (delaying flowering). Across haplotype blocks for all of the 13 circadian clock‐related genes that we could test, haplotype frequencies differed between cultivars and landraces, with a greater range of haplotype states revealed in the latter case (Figure S9). This was also reflected in an exome‐wide analysis of haplotype states, which demonstrated that the least common states were more often observed in landraces than in cultivars (Figure S10), indicating the value of landraces as sources of alleles for breeding.

### Latitudinal distributions of haplotypes at key circadian clock‐related genes indicate the importance of sampling contexts and barley category in understanding adaptive responses

**Figure 7:**
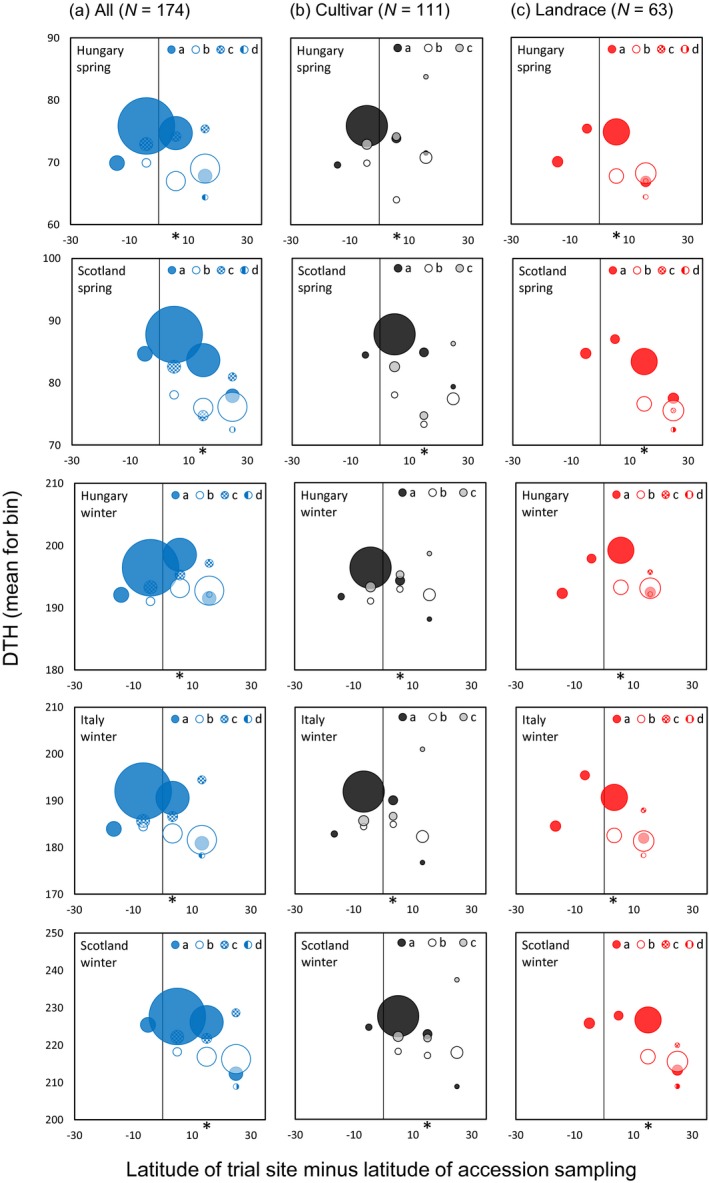
Mean days to heading (DTH) of specific *HvCEN* haplotype states across five environments for 174 spring habit domesticated barley lines.Each circle represents genotypes whose site of origin is within the same latitude bin of 10°. The *x*‐axis was centred by the latitude of each trial site (i.e. points to the right of the *x*/*y* intercept are south of the trial site). Genotypes were classified as (a) all genotypes, (b) cultivars or (c) landraces. The point size indicates the relative number of accessions with a particular *HvCEN* haplotype in a specific latitude bin. The asterisk (*) indicates the position of a mid‐latitude bin with clear differentiation in DTH for haplotypes *a* and *b* in all tested material.

We further explored the geographical origins of haplotype states at *HvCEN* and *HvPPD‐H1* genes with reference to the latitude of genotype sampling for our subset of 174 spring habit domesticated barley genotypes. Haplotype states for both genes followed a clear geographical gradient (Figures 7 and S11) that parallels earlier gene‐specific studies (Jones *et al*., 2008; Russell *et al*., 2016) and the wider geographical sampling of haplotype states for all 371 of our tested barley accessions (Figure S12). Most obviously for *HvCEN*, haplotype *a* dominates in northerly clines and *b* in more southerly locations. We also found a significant latitudinal effect consistent with the importance of the latitude‐based genic context of domestication‐related genes, and coinciding with the cultivar and landrace categories (Figures 7b,c and S11b,c). A comparison of haplotypes *a* and *b* in the results presented for *HvCEN* (Figure 7a), for example, indicated the key role for haplotype state in DTH variation for genotypes sampled from mid‐latitude points especially [haplotype *a* heading on average more than 7 days later than *b* (mean across five trial environments), which is also consistent with the QTL×E analysis]. Similar, although less clear, effects (possibly reflecting smaller sample sizes for the larger number of haplotype states for our subset of spring habit accessions) to those for *HvCEN* were observed for *HvPPD‐H1* (Figure S11). Results therefore reveal the importance of the genotype sampling context and history of breeding in understanding the adaptive responses of the barley crop, as these processes might lead to collinearities between alleles for different genes.

## Discussion

Our joint modelling of the barley crop's genotypes and multi‐environment phenotypes involved refined statistical approaches to analyse exomic gene haplotype states and explore environmental adaptation. Our assessment has built on previous research (Russell *et al*., 2016) by including a wider range of domesticated barley lines and by providing a more adaptive context to observed genotypic variation, through a formal multi‐environment analysis of adaptive phenotypes. Our analysis is of particular relevance for the development of the barley crop in important European production environments.

In our study, exomic variation that corresponded with barley classifications was distributed geographically in patterns consistent with an isolation by distance model (Wright, 1943), but contained specific features indicating selective processes at particular sequences, as demonstrated by circadian clock‐related genes. Even in the case of overall (rather than gene‐specific) genomic variation, however, local geographical components were not negligible, suggesting the presence of a degree of local sub‐structuring, probably due to more complex local climatic mosaics and perhaps reflecting ancient anthropogenic processes of landrace seed distribution. Taking the total exomic data and applying statistical methods for local LD characterization and clustering, we identified sub‐populations that explained an important proportion of phenotypic variance in the barley crop, with clear biological, geographical and historical interpretations. The large differences we observed in local LD patterns were consistent with other studies on plants, humans and animals (Dawson *et al*., 2002; Wang *et al*., 2013), and indicate the importance of local LD characterization in genetic research.

Classifications and phenotype–genotype comparisons, especially starting with exomic haplotype states that provided additional insights into diversity, revealed greater variation in landraces than cultivars and candidate gene loci supporting crop sub‐class identification and adaptive trait variation. They also quantified the relative importance of a panel of circadian clock‐related genes in determining genotype and G×E effects for the DTH trait. In addition, comparisons, as expected (Beaumont, 2005; Russell *et al*., 2016), revealed strong genetic differentiation for loci known to be responsible for determining barley's row classification (e.g. *Vrs1* and *INT‐C*) that confirmed the validity of our data, as did strong associations revealed by GWAS between known trait genes and our phenotypic field measurements.

Patterns of multi‐site phenotypic variation indicated clear interactions with genotypic data consistent with adaptation profiles that varied for spring and winter crop growth habits, and were consistent with the known history of crop development. Some differences in adaptability suggest that landraces in particular might be a useful source of alleles to breed for more broadly adapted cultivars. However, it is hard to make solid conclusions when comparing the cultivar and landrace categories on this point, as the comparison is partially confounded with row type and growth habit. The nature of interactions can be complex, with the directions of effects across trials varying for a number of clock‐related genes. In the case of the photoperiod response gene *HvPPD‐H1*, for example, particular haplotype states were associated with contrasting responses in spring‐sown and winter‐sown trials, while for the earliness per se gene *HvCEN* the second most common haplotype showed a consistent effect across all trials. The identification of further haplotypes that have an equivalent biological effect would be an additional step to characterize germplasm adaptation patterns and scope, with contrasting alleles in particular providing insights into the geographical regions that are promising sources of alleles useful for European crop breeding programmes. Contrasting groups can for example be used for crosses to efficiently search for causal genes (Ellis *et al*., 2000) and to provide breeders with climate‐ready alleles for inclusion in pre‐breeding initiatives. We also observed that known causal mutations in clock‐related genes (Turner *et al*., 2005; Comadran *et al*., 2012), especially for *HvCEN*, depended on a genic context defined by the latitude of origin of crop lines, as well as breeding history, for expressing phenotypic variation. This observation is consistent with theories of the release of large effects at crop domestication‐related gene loci with geographical range expansion from the Fertile Crescent to more northerly environments (Doust *et al*., 2014).

At a practical level, the large number of SNPs and their partitioning in our study has shown the extensive genetic diversity available in the WHEALBI germplasm collection for high‐resolution novel allele mining to support crop adaptation. This will allow the prioritisation of genomic regions and particular sets of lines for further inspection in crosses to support breeding efforts that seek to cope with climate change and a range of other abiotic and biotic stresses (Dawson *et al*., 2015).

An important motivation of our work was to provide not only genotypic and phenotypic data to the wider barley research community but also associated seed, and all are freely available through the WHEALBI project (http://www.whealbi.eu/). The suite of powerful refined statistical tools for multi‐environment GWAS and other analyses that we have developed here to address methodological challenges, including those posed by the significant genetic structuring in our germplasm panel (which is not unique to barley collections but occurs for many crops), are also freely available from the project website. Genotypes that are genetically similar because of population structure share both causal and non‐causal alleles, potentially leading to complex collinearities between different regions and spurious marker–trait associations that require correction (Korte and Farlow, 2013; Vilhjálmsson and Nordborg, 2013). In addition, the mixed model methodologies (Hoffman, 2013; Millet *et al*., 2016; Price *et al*., 2006) that we have used here are useful for providing insight into the genetic basis of adaptation across environments for crops other than barley. Finally, in the current study, we have focused on the analysis of exomic SNPs above a minimum minor allele frequency. Further research will explore lower‐frequency SNPs as well as indels and copy number variants to classify barley genotypes and for allele mining.

## Experimental Procedures

### Plant material

The domesticated barley germplasm employed for our genomic–multi‐environment phenomic comparison was taken from a wider sample of 512 accessions assembled by partners of the WHEALBI research project (EU FP7 no. FP7‐613556). This source material represents a range of worldwide barley genetic diversity, including landraces, cultivars and progenitors, assembled to quantify variation and explore adaptation, to determine possible breeding responses to production challenges (http://www.whealbi.eu/). In order to represent the geographical and environmental variation that reflects responses to biotic and abiotic stresses, the WHEALBI panel includes accessions originating from a wide range of locations covering key crop production regions in Europe, Africa, the Middle East and Asia. As explained further below, a subset of 371 domesticated lines was chosen from the entire WHEALBI germplasm set for current analysis, considering the completeness of both genomic and phenotypic data that allow detailed comparison. Chosen accessions covered landraces and cultivars (Figure S13) of both two‐ and six‐row types, and spring and winter growth habits [i.e. barleys that are generally planted in the spring and autumn (or winter), respectively, with cold‐tolerant winter barleys requiring vernalisation for timely flowering and crop production; Sasani *et al*., 2009]. Data on the origins of accessions are provided in Table S1. For some previously non‐located cultivars, we assigned geographical coordinate positions based on mid‐points of the main sub‐national barley production regions within their countries of origin, based on EUROSTAT (http://ec.europa.eu/eurostat/web/agriculture/data/) and other online data sources. Seed from each original genebank accession was multiplied through two rounds of single‐seed descent. One seed from the subsequent seed stock was germinated for DNA extraction, while most of the remainder was placed into the IPK genebank as reference material for future further testing and distribution (seed available on request from IPK). The final portion of the seed stock was further multiplied and then used to establish field trials.

### Collecting phenotypic data

Common garden trials were sown in Dundee (Scotland, winter and spring planting), Martonvasar (Hungary, winter and spring planting) and Fiorenzuola d'Arda (Italy, winter planting only). Details of the locations of trial sites, with summary weather conditions during field experimentation, are provided in Table S4. Field trials followed an augmented partially replicated design. In each field trial, the full set of 512 accessions that make up the entire WHEALBI collection was sown in single plots, and in addition 102 accessions (almost 20%) had two replicates. Replicated lines were randomly drawn from the full accession set and therefore the complement used differed from one experiment to the next. To integrate results across experiments, two check cultivars were also included. These were the winter growth habit variety Meridian and the spring growth habit variety Irina, which were both recently released and are grown across Europe. To distribute checks evenly within each trial, each check was added to eight out of the ten incomplete blocks that were laid out following the principles of an alpha design. Eight of these blocks represented subdivisions of the full set of plots (512 accessions) being tested, while a further two blocks represented duplicated accessions. Each plot consisted of two rows of length 1.5 m, with 30 seeds planted per row. Plots were separated from each other by buffer rows of wheat. A sample of seed was collected from each plot at each site. A quality control phenotypic ‘grow‐out’ of each of these samples was made at KWS (Cambridge, UK). Ten accessions were excluded from the analysis owing to mismatches between the site samples.

The subset of key life history traits considered in the current analysis consisted of DTH, plant height, 1000‐grain weight and awn length. Collected phenotypic data are provided in Table S1. During trait measurement, heading was considered to occur when half of the heads had emerged for 50% of the plants in a plot (Z55 according to the Zadok growth scale). Plant height at maturity was measured from the base of the plant to the insertion of the spike for at least four plants per plot and values averaged. Grain weight was measured for three aliquots of seed that were oven‐dried at 60°C for 48 h. The 1000‐grain weight was calculated by dividing the aliquot weight by the number of grains and averaging across aliquots. Awn length was measured for at least four spikes per plot and values averaged. Adjusted means (Table S1) and generalized heritabilities (Table S5) were calculated using the R package *SpATS*, fitting a model using two‐dimensional (2D) penalised splines (P‐splines) for the correction of spatial trends (Rodríguez‐Álvarez *et al*., 2018; Velazco *et al*., 2017).

### Library preparation and sequencing

Genomic DNA (gDNA) was extracted from leaf material of single barley individuals grown in the laboratory at IPK. Genomic DNA samples were checked with a Genomic DNA ScreenTape on the Agilent 2200 Tape Station System (Agilent, https://www.agilent.com/) to verify integrity. Samples were quantified by the Picogreen assay (Thermo Fisher, https://www.thermofisher.com/) and normalised to 20 ng μl^–1^ in 10 nm TRIS‐Hcl (pH 8.0) according to the NimbleGen SeqCap EZ Library SR protocol v.4.0. Genomic DNA was then fragmented to a size range of 180–200 bp using Covaris microTUBEs and a Covaris S220 Instrument (Covaris, https://covaris.com/), and whole‐genome libraries prepared according to the Kapa LTP Library Preparation protocol. Libraries were quantified using the Nanodrop method (Thermo Fisher) and analysed electrophoretically with an Agilent 2200 TapeStation System using a D1000 ScreenTape. Libraries were pooled in 8‐plex and hybridised with the barley SeqCap EZ oligo pool (Design Name 120426_Barley_BEC_D04; Mascher *et al*., 2013) in a thermocycler at 47°C for 48–72 h. Capture beads were used to pull down the complex of capture oligos and gDNA fragments, with unbound fragments removed by washing. Enriched fragments were amplified by PCR, and the final library quantified by quantitative (q)PCR and visualised using the Agilent TapeStation. Sequencing libraries were normalised to 2 nm, denatured with NaOH and used for cluster amplification on the cBot. The clustered flow cells were sequenced on an Illumina HiSeq2000 with an 8‐plex strategy (i.e. eight samples per HiSeq lane), with a 100‐bp paired‐end run module, at Parco Tecnologico Padano, Italy.

### Sequence processing and alignment

Sequence quality control was performed with FastQC (Babraham Institute, http://www.bioinformatics.babraham.ac.uk/projects/fastqc/). Raw Illumina reads were then trimmed with Trimmomatic v.0.30 (Bolger *et al*., 2014) to remove sequencing adapters and quality filtered using a sliding window of four bases, requiring a Phred quality score of >20. Trimmed reads were mapped to the reference genome (Mascher *et al*., 2017) with BWA v.0.7.5a, using the MEM algorithm with default parameters (Li & Durbin, 2009). A total of approximately 24 million reads per sample were mapped to the reference genome. The resulting BAM files were sorted with Samtools (http://samtools.sourceforge.net/) and duplicated reads marked using Samblaster (Faust and Hall, 2014). Only properly paired reads longer than 50 bp were used in further processing. All bioinformatic analyses were performed using Pipengine (Strozzi and Jean Pierre Bonnal, 2017).

### Single nucleotide polymorphism calling, validation and imputation

Variant calling and realignment around indels were performed with GATK v.2.7.4 (https://www.broadinstitute.org/gatk/) following best practices. Final BAM files were combined using GATK UnifiedGenotyper, with default parameters and a minimum base quality of 30. Raw variant calls were initially hard filtered by requiring QD >30.0, MQ >40.0 and sample DP ≥10. High‐quality exome sequence data were obtained for 403 of a starting set of 512 WHEALBI genotypes. Where exome data quality was too low (for the balance of 109 genotypes), exome capture was later repeated. These repeat data are available in archival records but were not included in our current analysis. Single nucleotide polymorphisms taken forward for further consideration at this stage had to pass filtering criteria of ≥80% of genotypes represented, a quality score of >30 and ≥98% of all scores in the homozygous condition. Validation to confirm the identity of exome‐captured accessions consisted of comparing SNPs obtained from exome capture with SNPs independently obtained with GBS and a SNP array. Data were consistent for the 403 lines reported here.

From the 403 initial genotypes for which we had high‐quality exome data, current analysis focused on 371 domesticated genotypes, where we excluded 22 wild barley lines (identified according to passport data) and another 10 genotypes for which an alternate (from expectation) row‐type status during phenotyping was revealed or initial testing of SNP data revealed accession profiles that were not consistent with a domesticated status.

For the purposes of the current analysis we applied a further filtering criterion to SNPs by only including markers with a MAF of ≥0.05. This was because our primary concern in the current study was to focus on constructing ‘basal’ haplotypes with higher frequencies more amenable for statistically valid phenotype–genotype comparisons. Our further analysis of the frequency spectra of SNP markers for landrace and cultivar categories, including lower‐frequency markers, showed that these spectra were fairly similar (although with landraces having a somewhat great proportion of very low‐frequency markers; Figure S14); thus our choice to apply a relatively stringent MAF should not unduly bias SNP inclusion toward either landrace or cultivar categories that could then very markedly influence relative diversity parameters (although it does of course ‘underestimate’ total diversity). Future allele mining and further assessment of diversity levels will involve analysis of SNP profiles without application of MAF.

For our set of 371 chosen accessions, absent SNP entries based on a MAF ≥0.05 were imputed with Beagle, implemented in the R package *Synbreed* (Wimmer *et al*., 2012). The SNP data set that included imputed SNPs was used for all subsequent analyses. These data were deposited at the Plant Genomics and Phenomics Research Data Repository of IPK and can be accessed via https://doi.org/10.5447/ipk/2019/5 (WHEALBI Consortium, 2019). The DOI was registered in the Plant Genomics and Phenomics Research Data Repository (Arend *et al*., 2016) with e!DAL (Arend *et al*., 2014).

### Measuring local LD

Patterns of meiotic recombination are known to be highly heterogeneous along barley chromosomes, with, for example, large peri‐centromeric regions virtually devoid of genetic crossing over (Mascher *et al*., 2017). In the current study we therefore developed and applied a method that characterizes local LD along chromosomes, using markers thinned to every 25th SNP. The method was employed twice, first without and then with kinship correction having been applied to the data (Mangin *et al*., 2012).

#### Method 1: LD characterization without kinship correction

A sliding window of 500 thinned SNPs was used to calculate local LD for each SNP position, employing the R package *LDcorSV* (https://cran.r-project.org/package=LDcorSV). Consecutive sliding windows had an overlap of 475 out of the 500 SNPs. Subsequently, SNPs within a distance of 20 000 bp were binned and the 0.95 quantile for the LD calculated for each bin. A monotonically decreasing spline was fitted to these binned SNPs and used to estimate the distance at which *R* ^2^ decreased to levels of 0.1 or 0.3 (these thresholds were applied because they correspond with those commonly used in the literature). Splines were then fitted with the R package *mcgv* (Wood, 2017). Distances characterizing the LD decay along the genome were then used to define the number of SNPs used for kinship sub‐population construction (see below), so that genomic regions where LD decayed more quickly by physical distance were sampled more intensively than regions with slower LD decay, allowing us to more effectively discriminate genotypes (Speed *et al*., 2012).

#### Method 2: LD characterization with kinship correction

Using *R* ^2^ as a measure of LD assumes that its extent around a causal polymorphism depends only on a drift–recombination process in a random‐mating population without selection (Mangin *et al*., 2012). As this assumption does not hold in the case of diversity panels such as that used here, LD was also characterized after correcting for population structure, based on a kinship constructed with SNPs sampled taking LD decay into account (as related further elsewhere in Experimental procedures). In this second approach, we again used a sliding window of 500 thinned SNPs. Calculation of kinship‐corrected LD followed the method proposed by Mangin *et al*. (2012) and was implemented in the R package *LDcorSV*. As for LD calculations without correction for population structure, we binned SNPs and fitted a monotonically decreasing spline to bins, and then used this to estimate the distance at which *R* ^2^ decreased to 0.1 or 0.3. Our purpose for calculating LD estimates after applying kinship correction was to gain insights into changes in LD along chromosomes that were independent of otherwise‐confounding population structure, as this knowledge is useful for a number of purposes, including determining the width of QTL regions.

### Characterizing population structure and genetic differentiation

To characterize population structure, 12 819 SNPs were sampled genome wide after local LD (not corrected for population structure, see above) had been taken into account. To define the number of sampled SNPs, all SNPs were first assigned to bins of 2 Mbp. Five SNPs were then sampled from the bins with the highest LD, but for bins with more rapid LD decay the number of SNPs sampled was increased proportionally to give greater weight to those genomic regions that provide for better genotype discrimination (Speed *et al*., 2012). Total sampled SNPs were then used to calculate a kinship matrix with the equation proposed by Astle and Balding (2009), implemented in the R package *Synbreed* (Wimmer *et al*., 2012). To infer the number of sub‐populations, significant principal components were calculated after applying a spectral decomposition to the kinship matrix (Patterson *et al*., 2006). We then assigned genotypes to sub‐populations using a hierarchical clustering procedure that was applied to the significant principal components, following Odong *et al*. (2013). Values of genetic differentiation (*F*_st_), based on row type (two‐rowed versus six‐rowed) and breeding history (landrace versus cultivar) were also calculated, using the R package *pegas* (Paradis, 2010). Differentiation was calculated for each SNP and moving medians of values were then obtained for windows of 100 SNPs using the R package *zoo* (Zeileis and Grothendieck, 2005).

### Landscape genomic analysis

We identified a well‐defined subset of spring growth habit barleys (*n *=* *174, comprising 111 cultivars and 63 landraces) from our initial set of 371 domesticated accessions for which the confounding factor of vernalisation in determining DTH had been removed by excluding winter growth habit lines. The genotypes chosen for this panel were non‐tropical, such that day length adaptation at specific latitudes should be an important feature for the crop. All the chosen genotypes also had collection site coordinate data. We use this panel to analyse some of the geographical details of control of DTH by specific gene sequences. Initially, however, we subjected the panel to a landscape genomics analysis using sPCA (Jombart *et al*., 2008), implemented in the R package *adegenet*, to determine the overall pattern of geospatially related genetic structure in the spring habit crop. In this method, the pattern of genomic variation is characterized in relation to spatial data using a matrix *X*, with dimensions genotype (individual) by marker (SNP). Spatial information contained in a degree distance matrix ***L***, standardized by rows and with diagonal terms set as zero, was used to calculate the spatial autocorrelation of the SNP alleles using Moran's *I*:

(1) $$I\left(x\right)=\frac{X^{T}LX}{X^{T}X}$$

Moran's *I* can be used to measure spatial structure in the values of *X*: it is highly positive when values of *X* observed at neighbouring sites tend to be similar (positive spatial autocorrelation, referred to as global or regional structures), while it is strongly negative when values of *X* observed at neighbouring sites tend to be dissimilar (negative spatial autocorrelation, referred to as local structures). Moran's index measures only spatial structures and not genetic variability, because it is standardized by the variance of *X*. Spatial structure and variation can be estimated in sPCA as

(2) $$C\left(X\right)=var\left(X\right)I\left(X\right)=\frac{1}{n}X^{T}LX$$

*C*(*X*) is highly positive when *X* has a large variance and exhibits a global structure. *C*(*X*) is negative when *X* has a high variance and displays a local structure.

### G×E characterization and genome‐wide association scans

G×E patterns were studied for the 371 domesticated barley lines with high‐quality genotypic and phenotypic data. Days to heading, plant height, 1000‐grain weight and awn length were evaluated with various genotype to phenotype models, focusing on the genotypic response across environments. Models will be presented here by increasing complexity:

(3) $$y_{\mathrm{ij}}=\mu +E_{j}+G_{i}+\text{G}{\text{E}}_{\mathrm{ij}}+\epsilon_{\mathrm{ij}}$$

In Equation  (3), *y*_*ij*_ is the phenotype for genotype *i* in environment *j*, μ is the intercept, *E*_*j*_ is the fixed effect of environment *j*,* G*_*i*_ is the random effect of genotype *i*, GE_*ij*_ is the random G×E interaction and ɛ_*ij*_ is the residual. To quantify the contribution of genotype classifications to the phenotypic variance, model (3) was expanded as follows:

(4) $$y_{\mathrm{ij}}=\mu +E_{j}+C_{k}+\text{C}{\text{E}}_{\mathrm{jk}}+G_{i(k)}+\text{G}{\text{E}}_{ij(k)}+\epsilon_{\mathrm{ij}}$$

where *C*_*k*_ is the fixed effect of genotype category and CE_*jk*_ stands for the fixed category‐by‐environment effects. Model (4) was fitted four times considering sub‐populations (kinship group, as related further elsewhere in Experimental procedures), row type (two‐rowed and six‐rowed), breeding history (cultivars and landraces), growth habit (winter landraces, winter cultivars, spring landraces and spring cultivars) and the combination of breeding history and growth habit [‘BH and GH’ (spring cultivar, winter cultivar, spring landrace, winter landrace, spring facultative landrace)] as categories. To have an estimate of the importance of a suite of known candidate genes related to DTH, model (4) was also fitted, replacing *C*_*k*_ and CE_*jk*_ by the haplotype states of these genes. The contribution of categories (Figure 3) or of known genes related to DTH (Figure S8) to genotypic and G×E variance was quantified by calculating the reduction in *G*_*i*_ and GE_*ij*_ between models (3) and (4). To further characterize adaptation patterns, Finlay–Wilkinson, GGE and AMMI models were used.

(5) $$y_{\mathrm{ij}}=\mu +G_{i}+\beta_{i}E_{j}+\epsilon_{\mathrm{ij}}$$

In the Finlay–Wilkinson model (Equation 5; Finlay and Wilkinson, 1963), the genotype main effect *G*_*i*_ provides an indication of general performance across all environments, β_*i*_ is the genotypic sensitivity to the environment and *E*_*j*_ is a covariable that characterizes the environmental quality and corresponds to the genotypic mean in environment *j*. Intercepts and slopes were compared for genotypes belonging to different categories using a one‐way ANOVA and a Tukey test.

To characterize adaptation patterns of the groups of genotypes across environments, the GGE interaction model was fitted (Yan *et al*., 2000; Yan and Kang, 2002).

(6) $$y_{\mathrm{ij}}=\mu +E_{j}+\sum_{p=1}^{P}r_{\mathrm{pi}}c_{\mathrm{pi}}+\epsilon_{\mathrm{ij}}$$

In Equation (6), $\sum_{p=1}^{P}r_{\mathrm{pi}}c_{\mathrm{pi}}$ represents the *P* bilinear terms that are used to model (*G*_*i*_ + GE_*ij*_).The first two bilinear terms are used to create a visual representation of the adaptation patterns (Yan and Kang, 2002). The AMMI model was also used to compare the contributions of genotype categories to the DTH G×E:

(7) $$y_{\mathrm{ij}}=\mu +G_{i}+E_{j}+\sum_{a=1}^{A}r_{\mathrm{ai}}c_{\mathrm{aj}}+\epsilon_{\mathrm{ij}}$$

In model (7), *A* bilinear terms are used to represent GE_*ij*_. The scores for the first two bilinear terms were compared for the different genotype categories using a one‐way ANOVA and a Tukey test.

Further investigation of the structure of G×E and its underlying genetic basis involved multi‐environment GWAS applying equation  (8), which was fitted with ASREML‐R (VSN‐International, 2015). This model is similar to those applied by Millet *et al*. (2016) and Thoen *et al*. (2017). The model addresses the analysis of multi‐environment data with the identification of genomic regions related to differential QTL expression across environments (QTL×E).

(8) $$y_{\mathrm{ij}}=\mu +E_{j}+\sum_{p=1}^{P}\left(x_{\mathrm{ip}}^{\mathrm{PC}}\beta_{p}^{G}\right)+\sum_{p=1}^{P}\left(x_{\mathrm{ip}}^{\mathrm{PC}}\beta_{\mathrm{jp}}^{\mathrm{GE}}\right)+x_{i}^{\mathrm{SNP}}\beta_{j}^{\mathrm{SNP}}+\text{G}{\text{E}}_{\mathrm{ij}}+\epsilon_{\mathrm{ij}}$$

In Equation  (8), *y*_*ij*_ is the phenotype of genotype *i* in environment *j*,* μ* is an intercept and *E*_*j*_ is the fixed effect of environment *j*. The terms$\sum_{p=1}^{P}\left(x_{\mathrm{ip}}^{\mathrm{PC}}\beta_{p}^{G}\right)$ and $\sum_{p=1}^{P}\left(x_{\mathrm{ip}}^{\mathrm{PC}}\beta_{\mathrm{jp}}^{\mathrm{GE}}\right)$ correct for population structure representing, respectively, the main effect population structure and the population structure by environment interaction. $x_{\mathrm{ip}}^{\mathrm{PC}}$ is the *p*th principal component extracted from the kinship matrix and $\beta_{p}^{G}$ is a regression slope indicating the importance of this principal component for the main effect population structure, while $\beta_{\mathrm{jp}}^{\mathrm{GE}}$ indicates the importance of this principal component for G×E. A specific kinship matrix was calculated for each chromosome when testing for marker–trait associations by excluding the markers on that particular linkage group (Rincent *et al*., 2014). The QTL/SNP effects are included via the term $x_{i}^{\mathrm{SNP}}\beta_{j}^{\mathrm{SNP}}$, with fixed environment‐specific SNP effects, $\beta_{j}^{\mathrm{SNP}}$, and marker information (allele counts) in $x_{i}^{\mathrm{SNP}}$. Thus, at each marker position, $x_{i}^{\mathrm{SNP}}\beta_{j}^{\mathrm{SNP}}$ models a QTL main effect and a QTL×E term simultaneously. Fitted QTL effects can be inspected and tested to identify QTLs that have a consistent effect across environments versus those that show QTL×E. Importantly, this latter category, related to adaptation, can be used to identify genotypes with adaptive alleles for specific production conditions. The significance of the QTL effect was assessed with a Wald test (Boer *et al*., 2007; Welham and Thompson, 1997). The significance threshold employed for testing marker–trait associations was established using a Bonferroni correction for independently segregating chromosome segments (Li and Ji, 2005) in combination with a correction for genomic control (Devlin and Roeder, 1999; Kang *et al*., 2008). The model in equation  (8) was used for testing QTL effects related to individual SNPs as well as for QTL effects related to haplotype blocks within gene regions (as described in the next section).

### Defining haplotypes

As input to the construction of haplotype blocks we used 37 535 candidate gene regions annotated with high confidence in the most recent version of the barley genome assembly (Beier *et al*., 2017; Mascher *et al*., 2017). For 18 278 genes that had multiple SNPs with MAF ≥0.05, we attempted to construct haplotype blocks and states with Haploview (Barrett *et al*., 2005) following the method of Gabriel *et al*. (2002). Sufficient LD was present to create haplotype blocks for 17 235 genes, with multiple blocks identified for some genes. To then explore the genetic basis for adaptation in a unique contribution to the methodology of genome‐wide association analysis, we developed a multi‐environment model that tested for haplotype block–trait associations on a genome‐wide scale, based on a total of 25 009 haplotype blocks. To this end, the SNP term $x_{i}^{\mathrm{SNP}}$ in equation  (8) above was replaced by a vector of haplotype states, $x_{i}^{H}$, while the allele substitution effect $\beta_{j}^{\mathrm{SNP}}$ was replaced by a vector of haplotype state substitution effects $\beta_{j}^{H}$ representing the effect of each haplotype state relative to the most common haplotype state (baseline reference).

## Data statement

The data that support the findings of this study are openly available in the Plant Genomics and Phenomics Research Data Repository at https://doi.org/, reference number 10.5447/IPK/2019/5.

## Conflict of interest

The authors declare that they don't have any conflict of interest.

## Author contributions

DBK carried out statistical analyses, drafted the first version of the manuscript and compiled co‐author inputs. IKD contributed to the work on gene interpretation and supported the compilation of co‐author inputs in the manuscript. JR conceived part of the project, and contributed to QTL and gene interpretation. AT undertook one of the multi‐environment field trials, and contributed to QTL and candidate gene interpretation. DG and NT contributed to the work on QTL and gene interpretation. CF performed library construction, exome capture and sequencing. FS and ELN performed SNP calling from exome sequences. MM‐L, HO, MM, PM, EC, EY and SD undertook multi‐environment field trials. SK contributed to the work on gene interpretation and helped develop data pipelines. AB contributed to the work on QTLs. DC used EUROSTAT and online data sources to gather information on sub‐national barley crop output and contributed to gene interpretation. MM contributed to the SNP calling and validation, and uploading the data to a public repository. PW contributed to the collection of phenotypic data and performed phenotype quality checks. LC coordinated the multi‐environment field trials, undertook one of the trials and contributed to the work on QTL and gene interpretation. LR coordinated exome sequencing and SNP calling and contributed to the work on QTL and gene interpretation. NS and BK assembled tested germplasm. RW conceived the project and edited the manuscript. FAvE proposed statistical models for analysis and edited the manuscript.

## Supporting information


**Figure S1.** Single nucleotide polymorphism (SNP) density, expressed as the number of SNPs per Mbp.

**Figure S2.** Relationship among genetic and geographic features for a subset of 174 spring habit domesticated barley genotypes.

**Figure S3.** Genetic differentiation (*F*_st_) between groups of barley genotypes.

**Figure S4.** Summary of phenotypic data for days to heading, plant height, 1000‐grain weight and awn length.

**Figure S5.** Genotype plus genotype by environment biplot for days to heading, plant height, 1000‐grain weight, and awn length.

**Figure S6.** Additive main effect and multiplicative interaction biplot for days to heading.

**Figure S7.** Manhattan plots based on haplotype states for days to heading, plant height, 1000‐grain weight and awn length.

**Figure S8.** Percentage of variance in days to heading for 371 barley genotypes explained by haplotype states for a suite of known circadian clock‐related genes.

**Figure S9.** Proportion of genotypes carrying each haplotype for circadian clock‐related genes.

**Figure S10.** Proportion of landrace genotypes, from all genotypes carrying the most and least common haplotype states.

**Figure S11.** Mean days to heading of specific *HvPPD‐H1* haplotype states across five field trial environments for 10° latitude bins.

**Figure S12.** Geographical distribution of haplotype states for clock‐related *HvPPD‐H1* and *HvCEN* genes.

**Figure S13.** Geographical distribution of cultivars and landraces.

**Figure S14.** Proportion of single nucleotide polymorphisms within minor allele frequency bins.

**Table S1.** Passport and phenotypic data for barley lines from the WHEALBI collection.

**Table S3.** Kinship matrix for 371 barley genotypes used for the genetic analysis.

**Table S2.** Number of single nucleotide polymorphisms (SNPs) per chromosome, with median and mean distances between SNPs for 403 barley genotypes characterized by exome capture.
**Table S4.** Environmental characteristics of field trials.
**Table S5.** Generalised heritability for days to heading, plant height, 1000‐grain weight and awn length.
**Table S6.** Variance components and standard errors for days to heading, plant height, 1000‐grain weight and awn length.
**Table S7.** Quantitative trait loci based on haplotype states for days to heading, plant height, grain weight and awn length.
**Table S8.** Additive effects across trial environments for a suite of circadian clock‐related genes involved in determining days to heading in the barley crop.
**Table S9.** Haplotype states for a suite of circadian clock‐related genes involved in determining days to heading in the barley crop, with their single nucleotide polymorphism alleles and frequencies.
