# Supplementary figures and images for: Exome sequences and multi‐environment field trials elucidate the genetic basis of adaptation in barley
Source: Plant J. 2019 Jun 27;99(6):1172–91. doi: 10.1111/tpj.14414 (PMC6851764; doi:10.1111/tpj.14414)

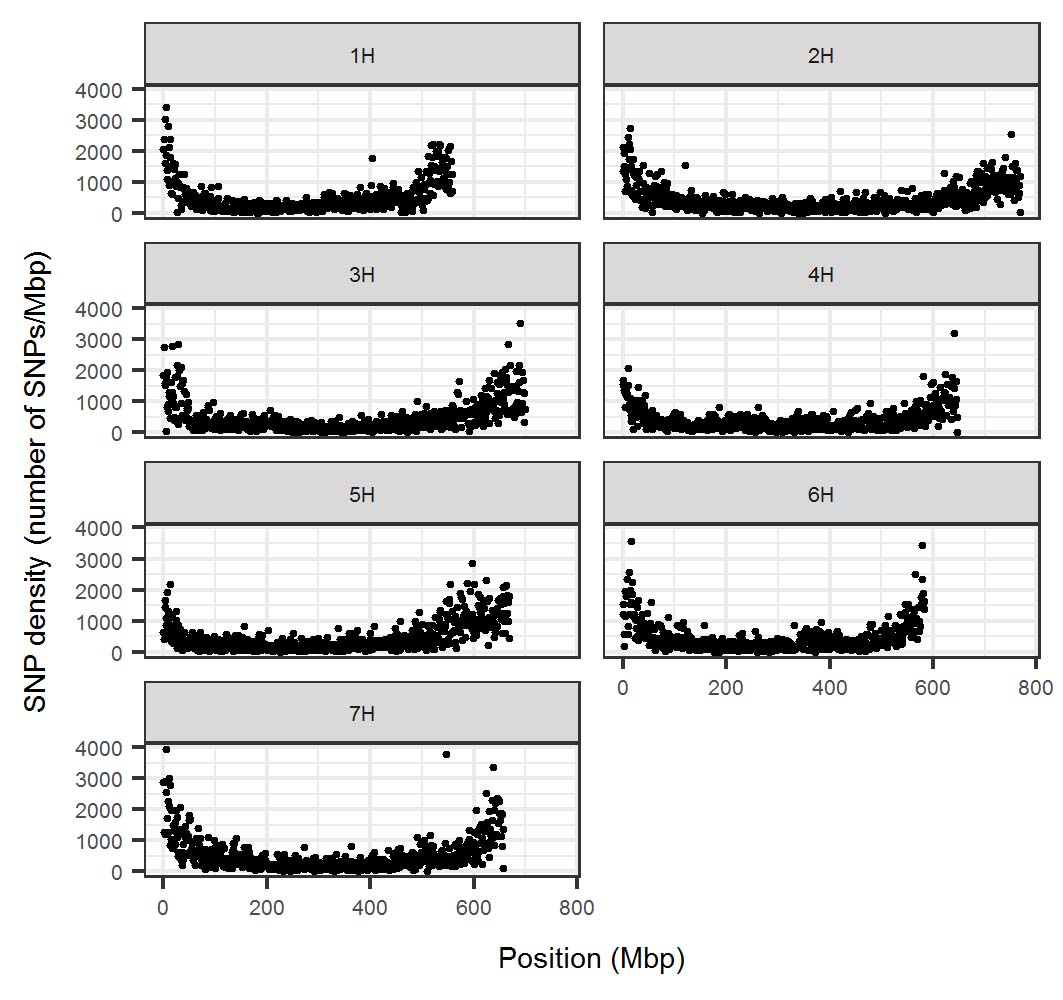

Supplement: Supplementary file 1 — Figure S1. Single nucleotide polymorphism (SNP) density, expressed as the number of SNPs per Mbp. [file TPJ-99-1172-s001.jpg]

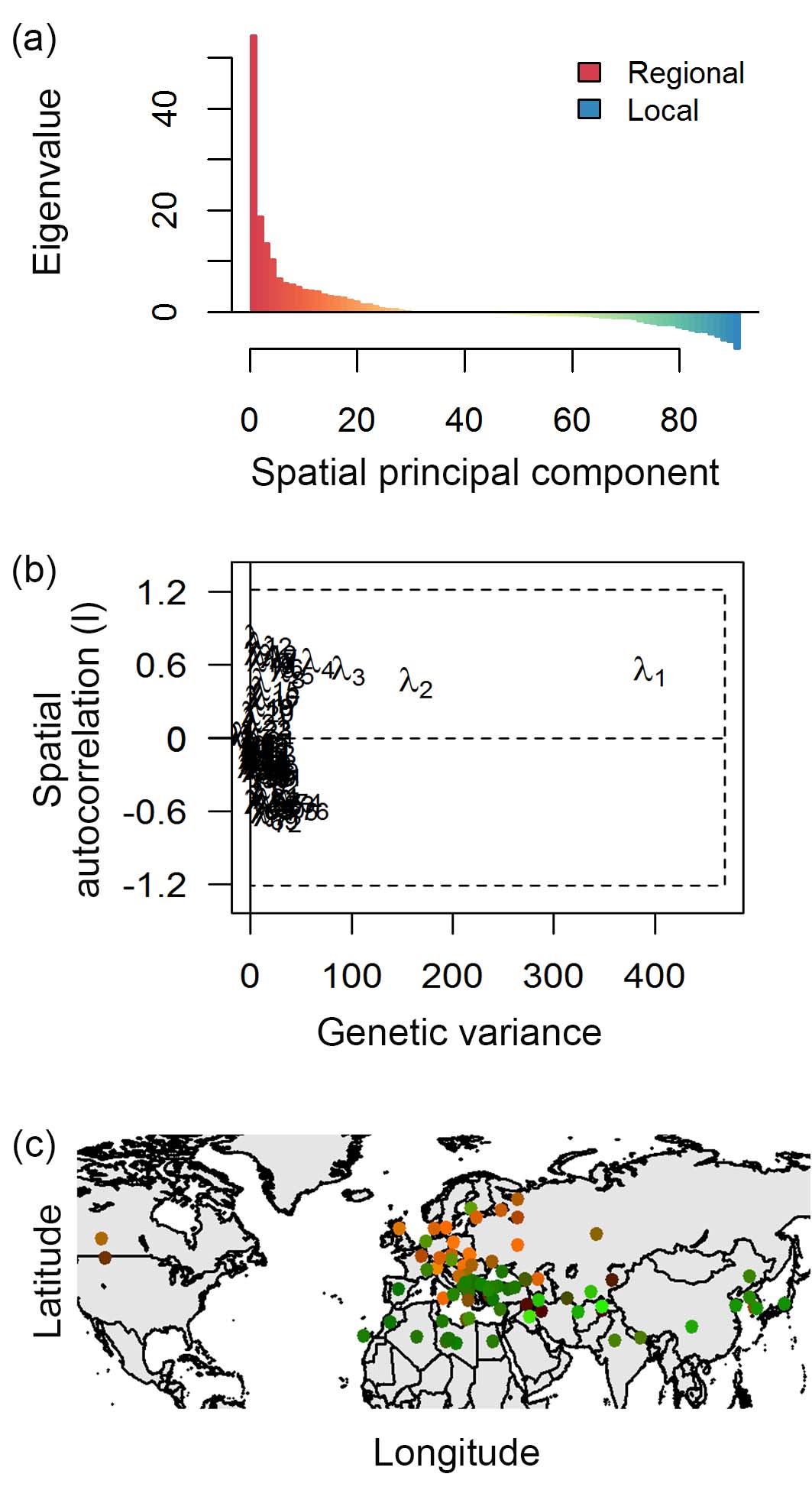

Supplement: Supplementary file 2 — Figure S2. Relationship among genetic and geographic features for a subset of 174 spring habit domesticated barley genotypes. [file TPJ-99-1172-s002.jpg]

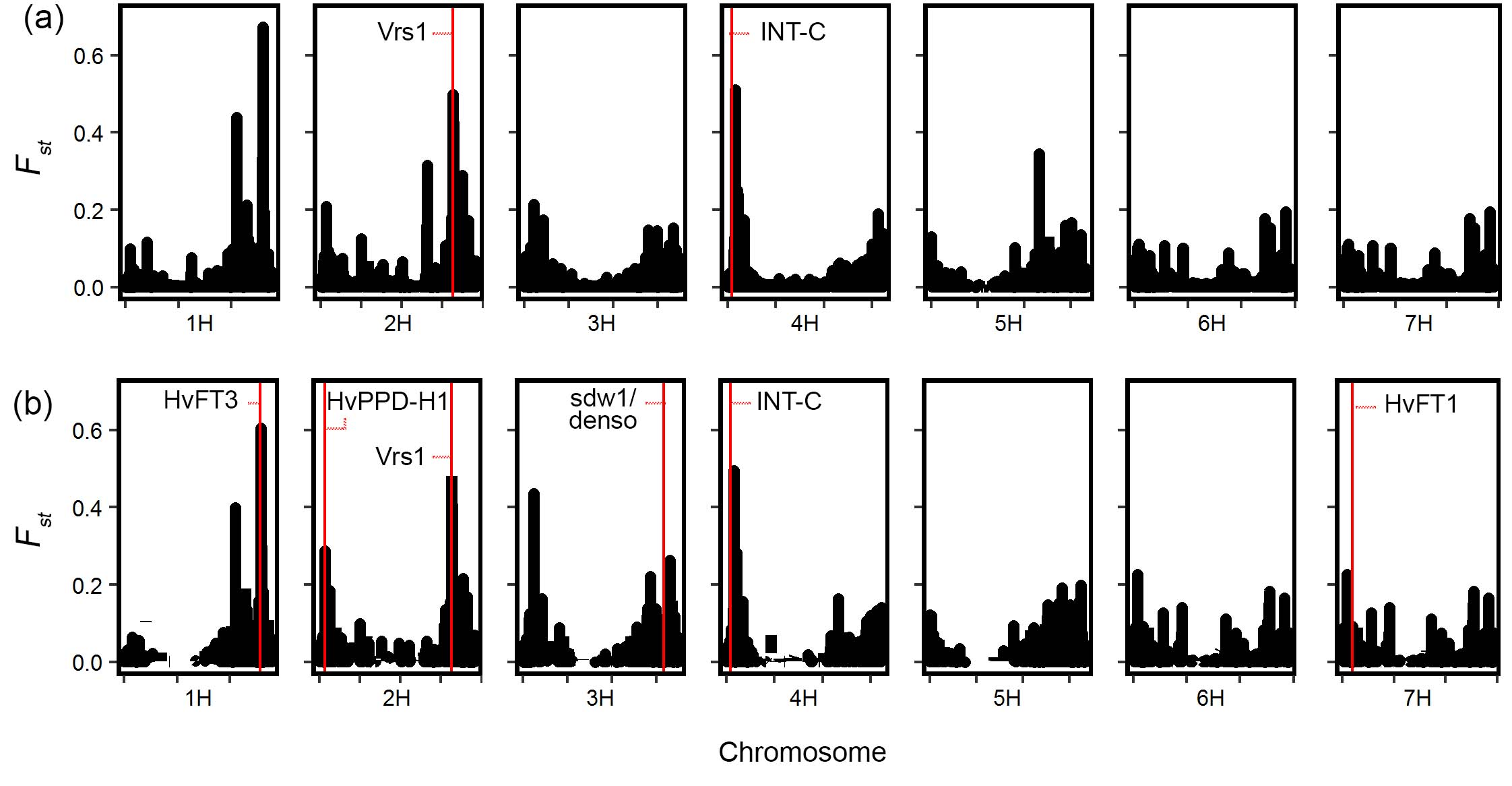

Supplement: Supplementary file 3 — Figure S3. Genetic differentiation (F st) between groups of barley genotypes. [file TPJ-99-1172-s003.jpg]

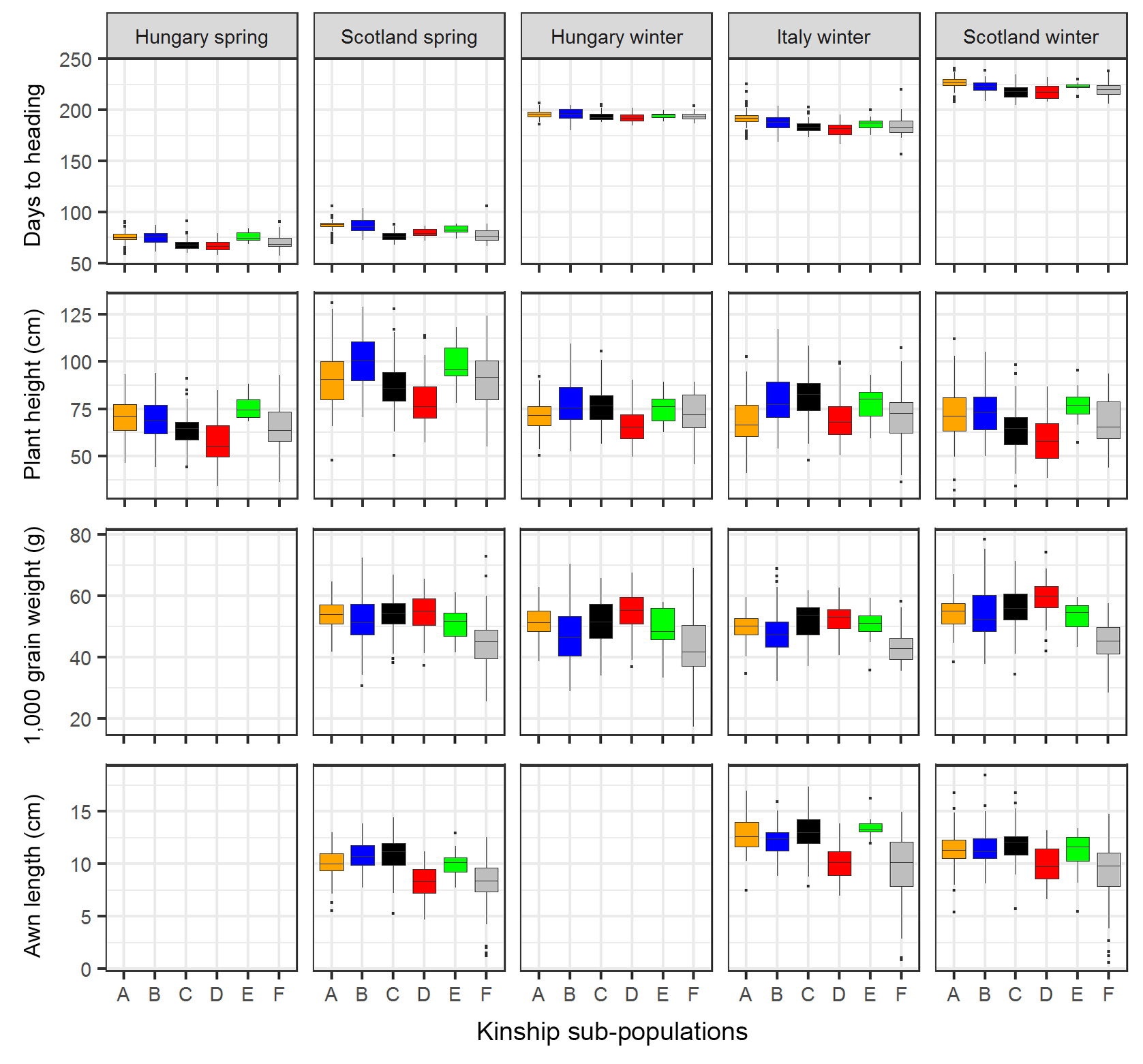

Supplement: Supplementary file 4 — Figure S4. Summary of phenotypic data for days to heading, plant height, 1000‐grain weight and awn length. [file TPJ-99-1172-s004.jpg]

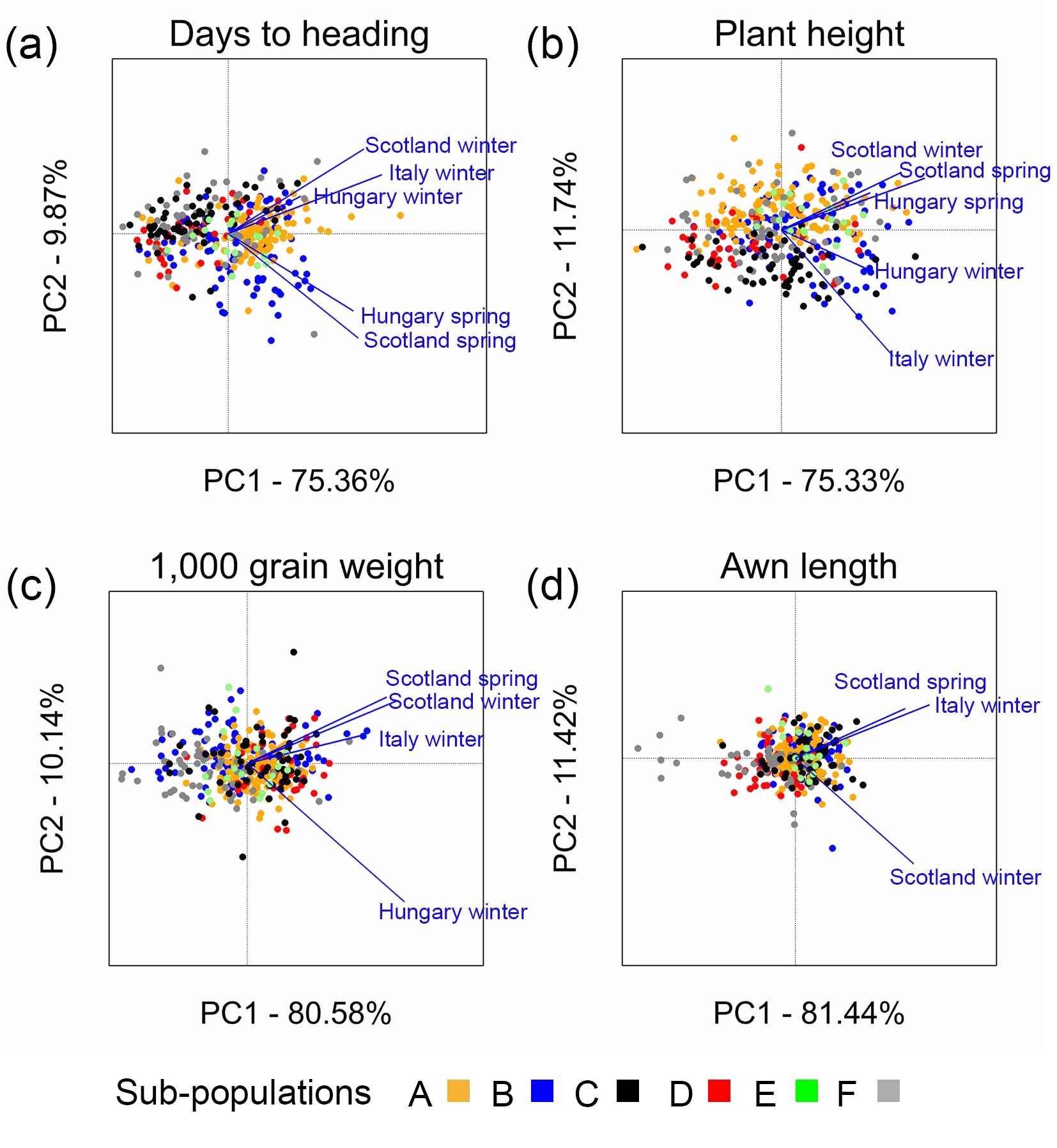

Supplement: Supplementary file 5 — Figure S5. Genotype plus genotype by environment biplot for days to heading, plant height, 1000‐grain weight, and awn length. [file TPJ-99-1172-s005.jpg]

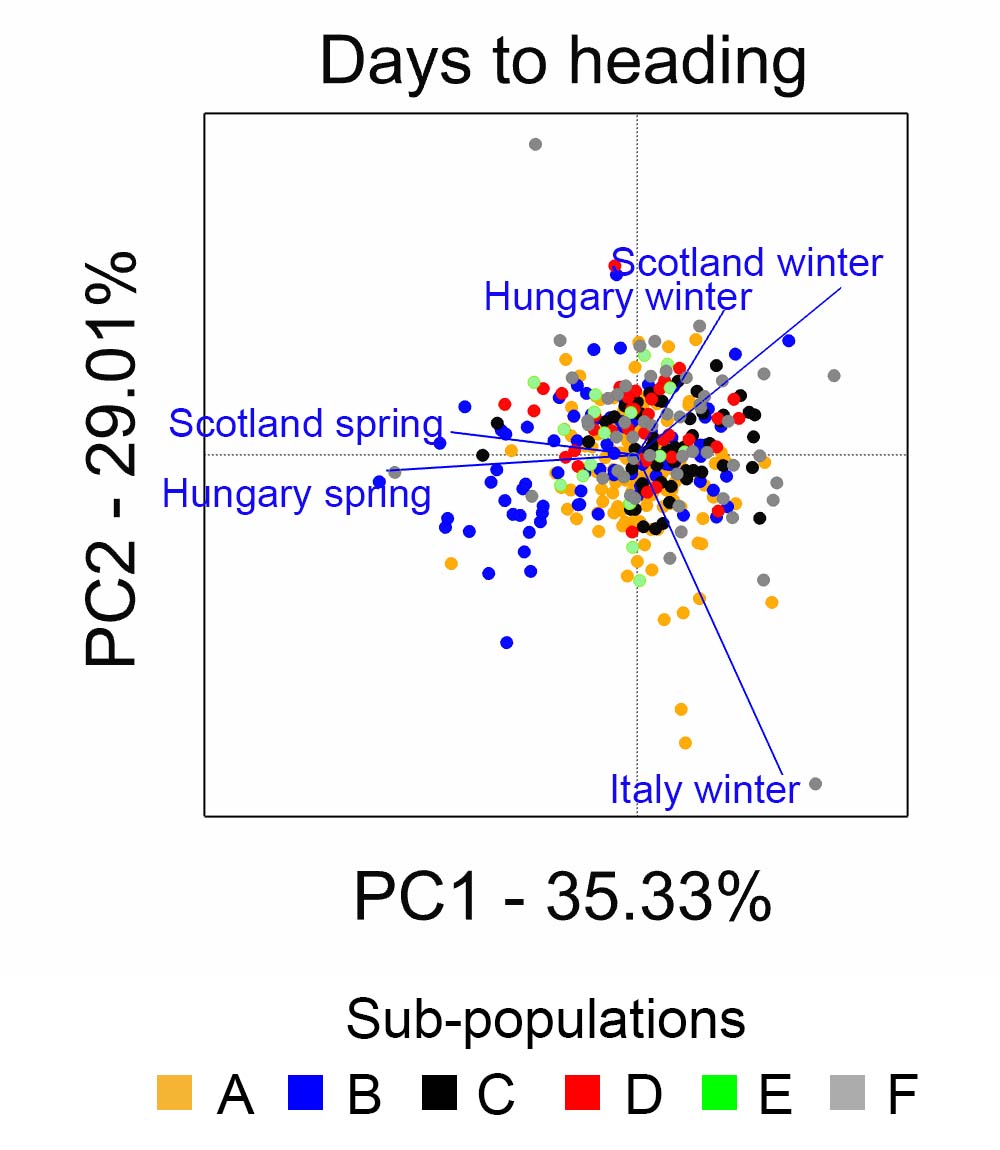

Supplement: Supplementary file 6 — Figure S6. Additive main effect and multiplicative interaction biplot for days to heading. [file TPJ-99-1172-s006.jpg]

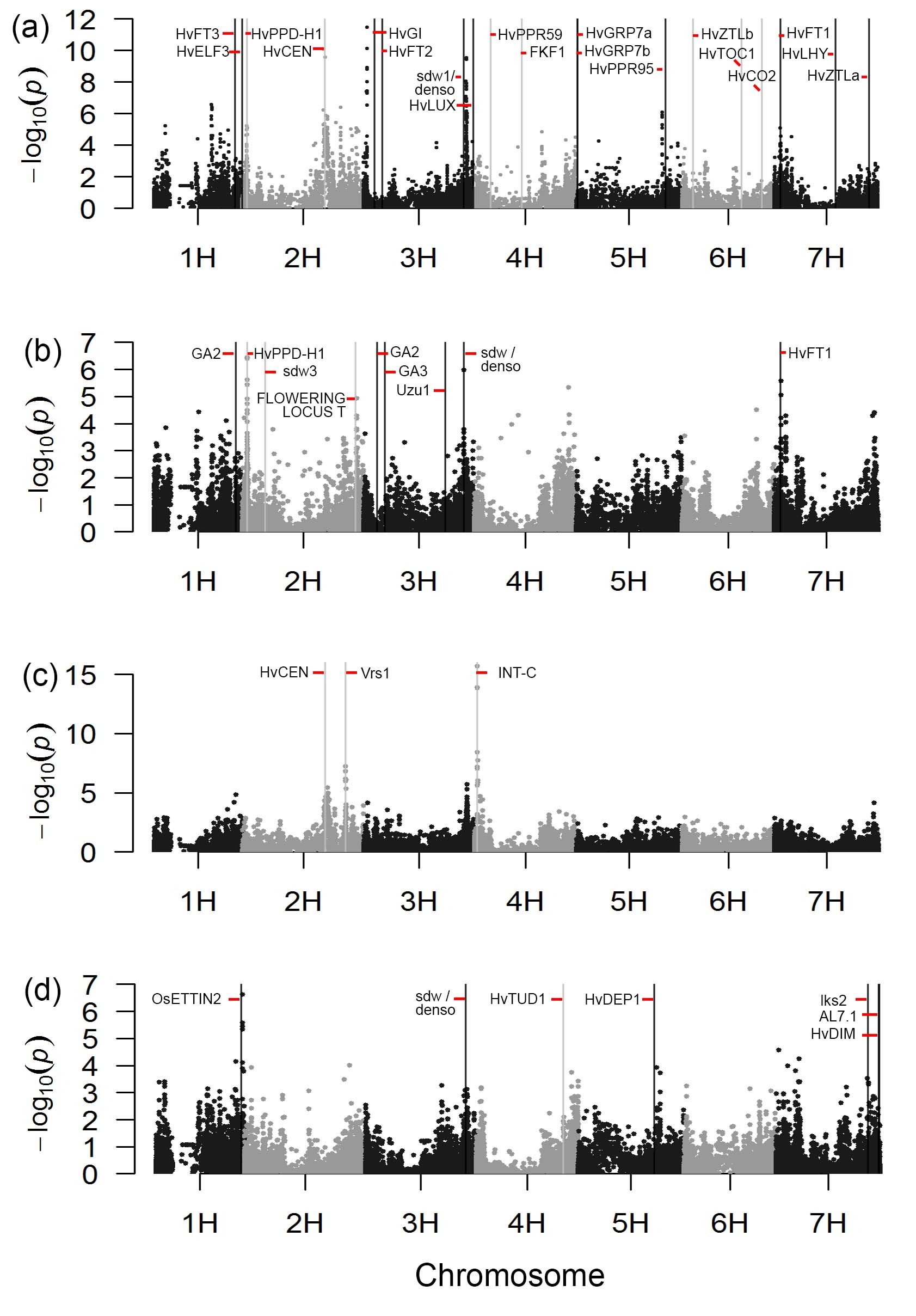

Supplement: Supplementary file 7 — Figure S7. Manhattan plots based on haplotype states for days to heading, plant height, 1000‐grain weight and awn length. [file TPJ-99-1172-s007.jpg]

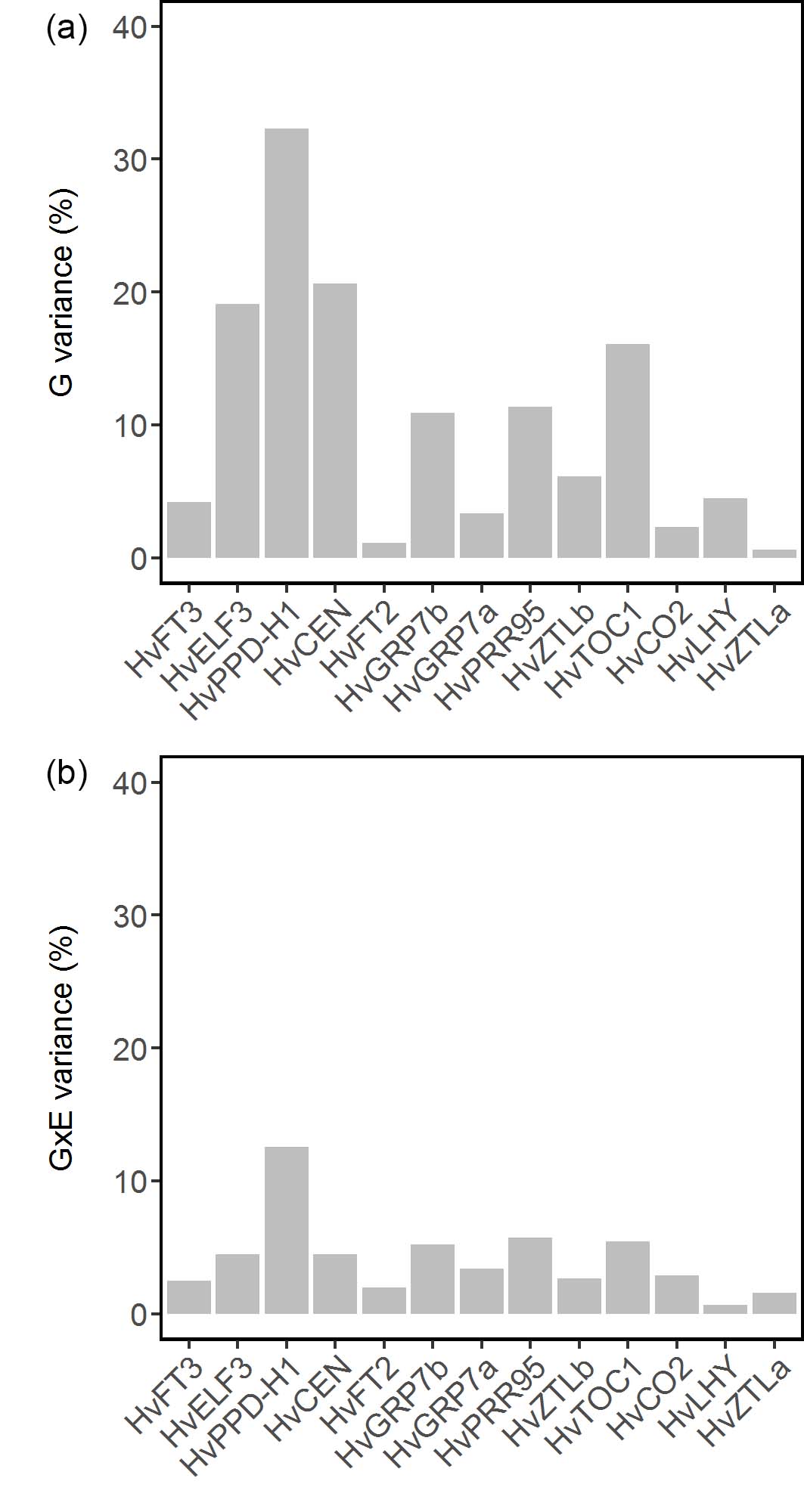

Supplement: Supplementary file 8 — Figure S8. Percentage of variance in days to heading for 371 barley genotypes explained by haplotype states for a suite of known circadian clock‐related genes. [file TPJ-99-1172-s008.jpg]

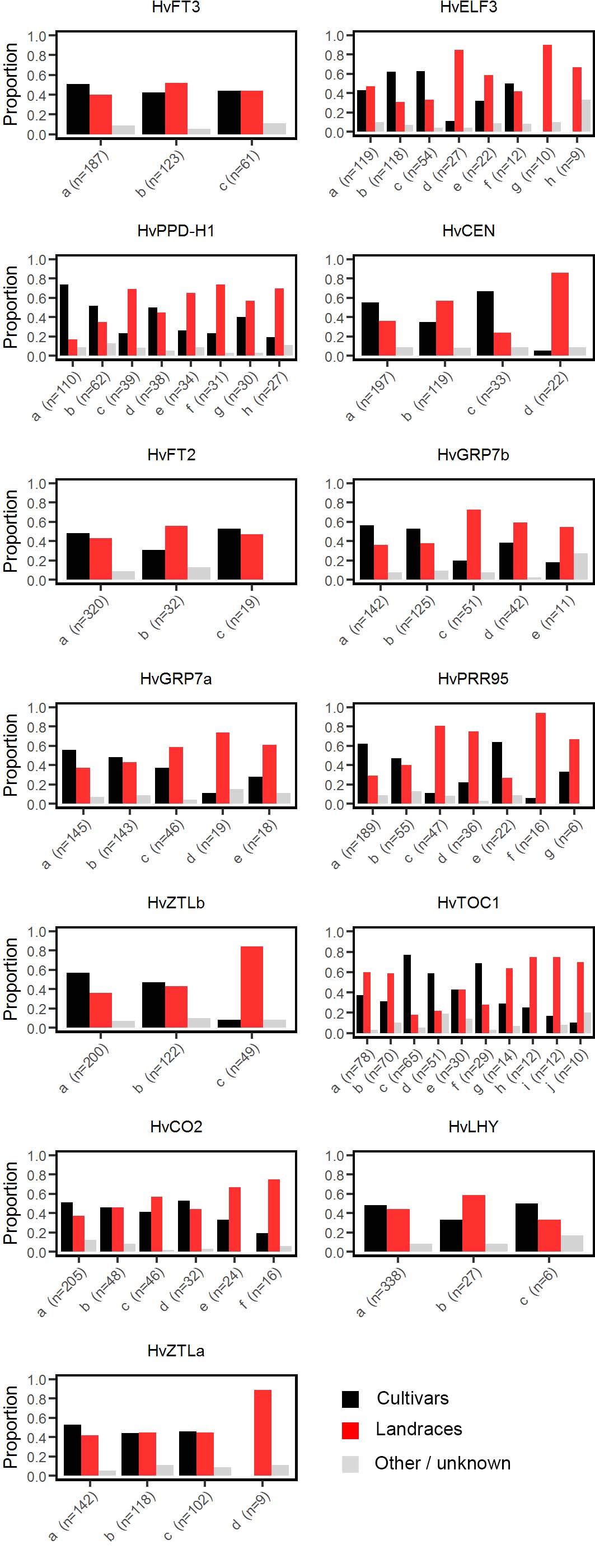

Supplement: Supplementary file 9 — Figure S9. Proportion of genotypes carrying each haplotype for circadian clock‐related genes. [file TPJ-99-1172-s009.jpg]

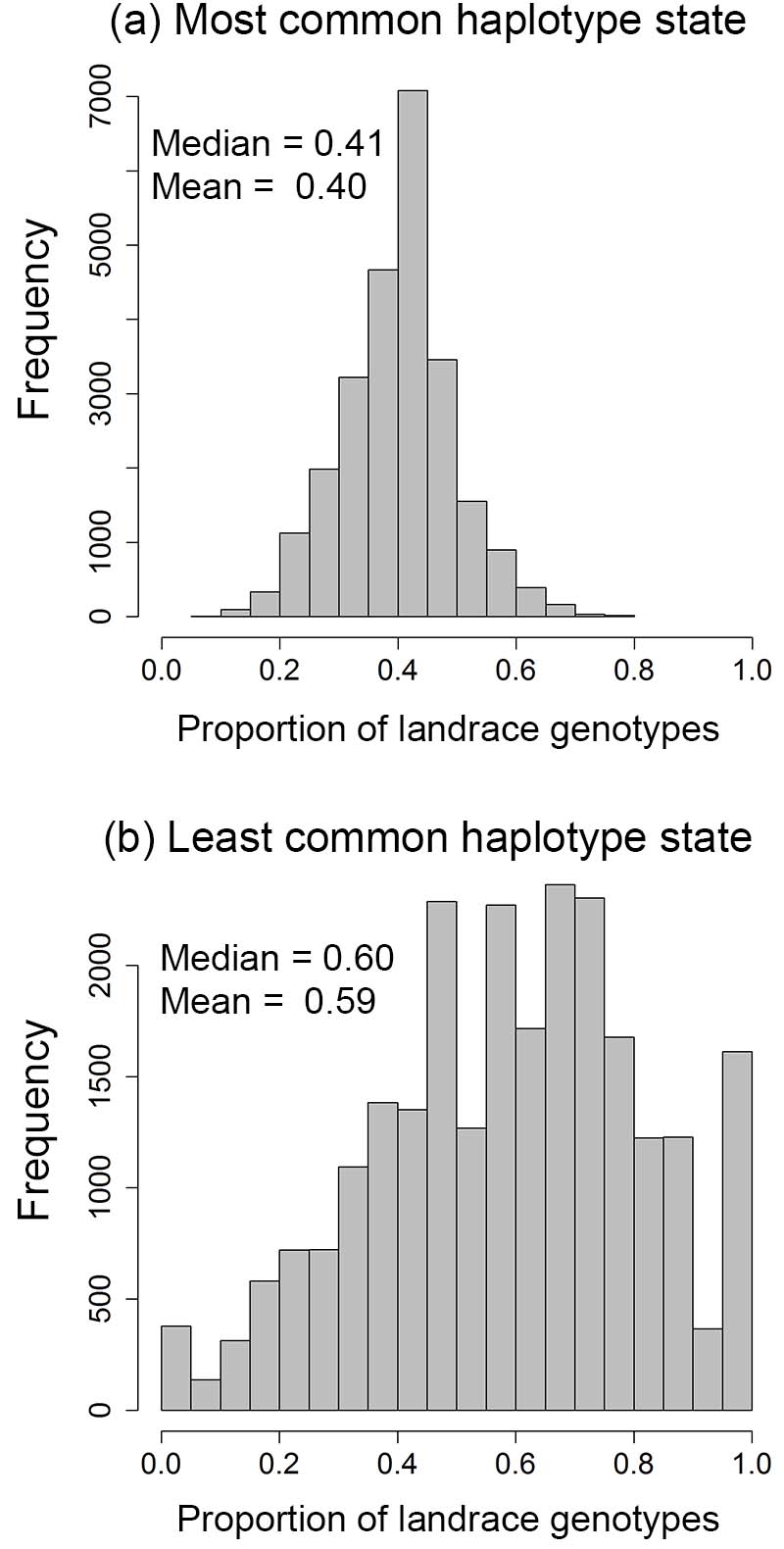

Supplement: Supplementary file 10 — Figure S10. Proportion of landrace genotypes, from all genotypes carrying the most and least common haplotype states. [file TPJ-99-1172-s010.jpg]

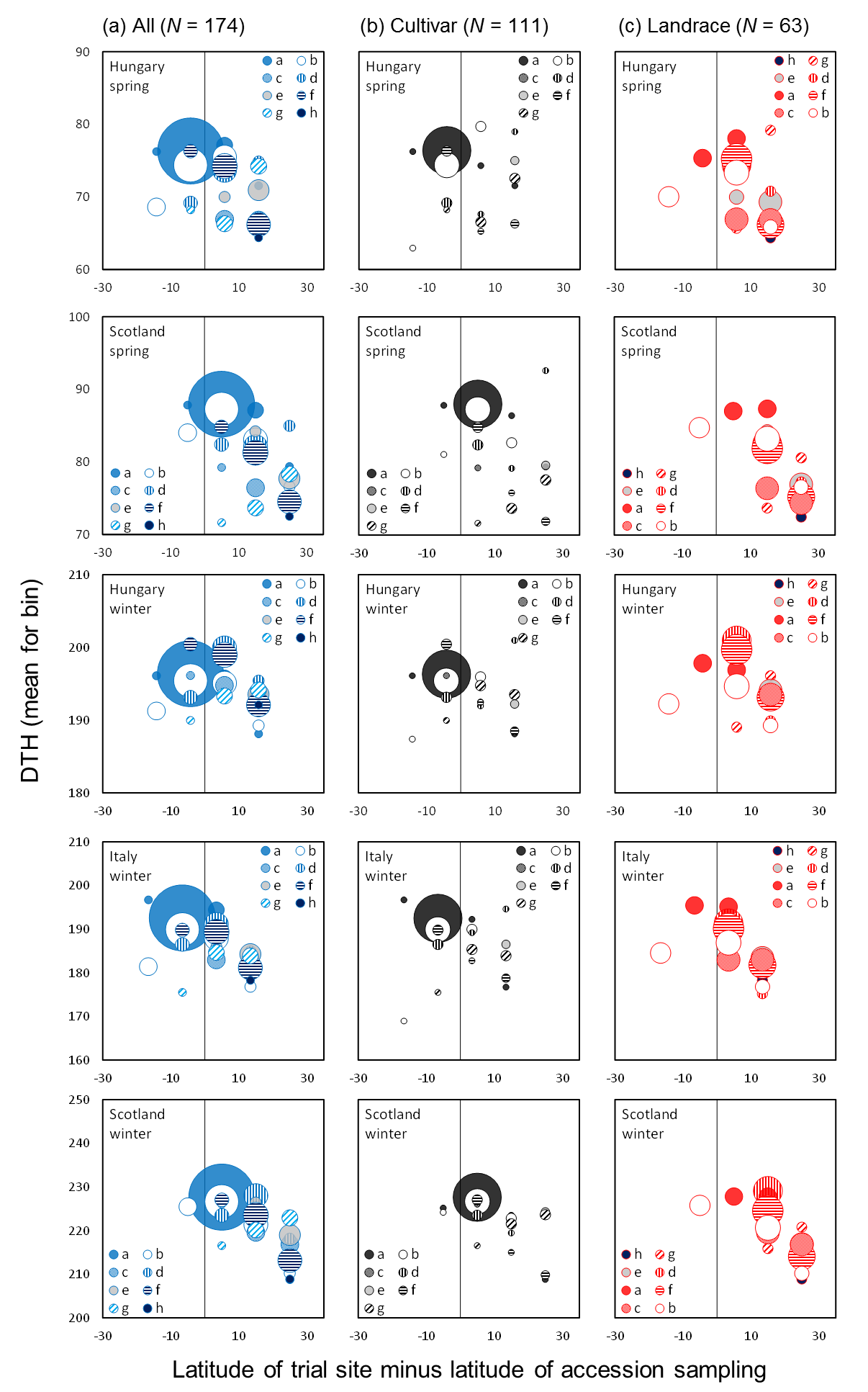

Supplement: Supplementary file 11 — Figure S11. Mean days to heading of specific HvPPD‐H1 haplotype states across five field trial environments for 10° latitude bins. [file TPJ-99-1172-s011.TIF]

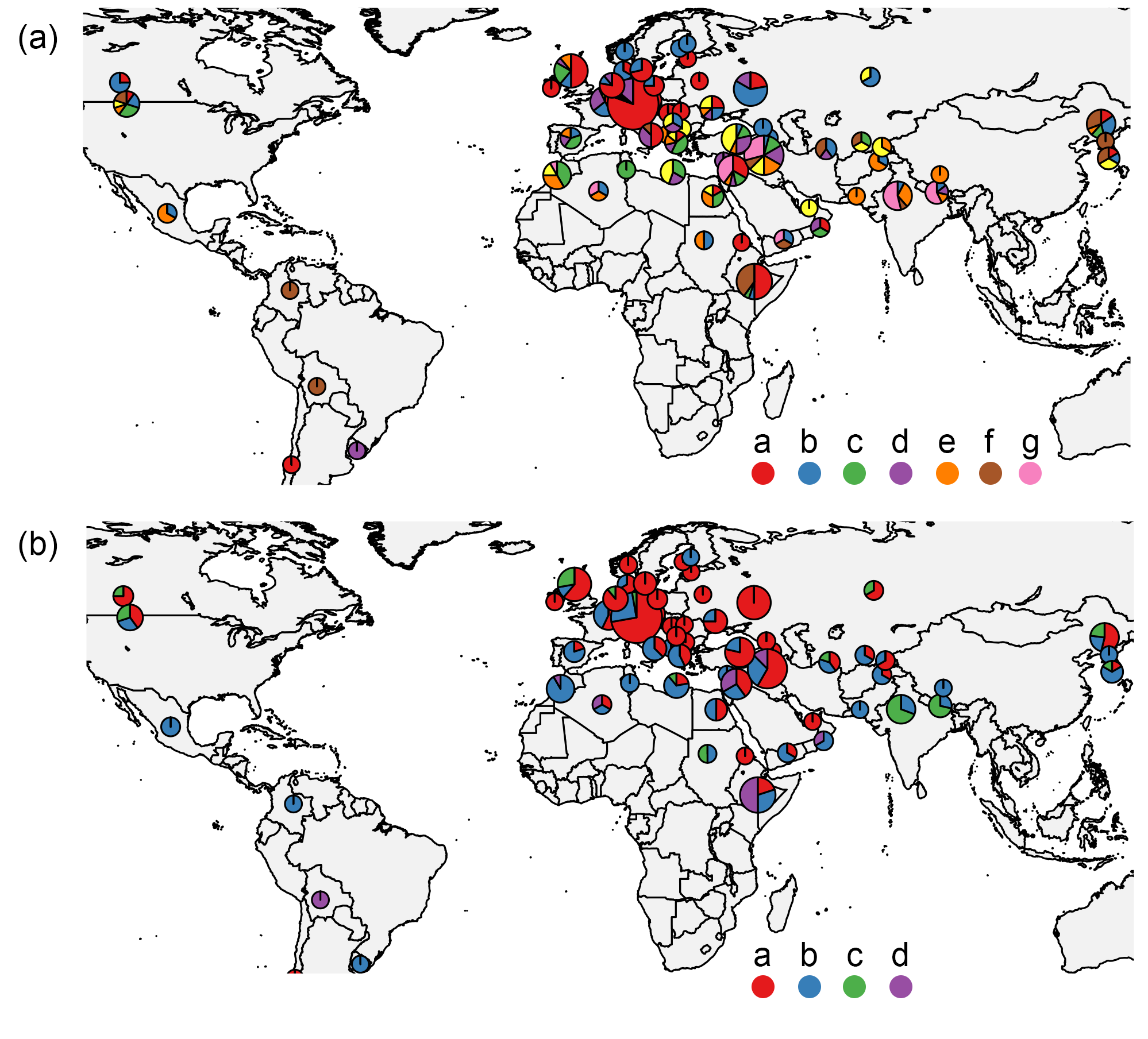

Supplement: Supplementary file 12 — Figure S12. Geographical distribution of haplotype states for clock‐related HvPPD‐H1 and HvCEN genes. [file TPJ-99-1172-s012.tif]

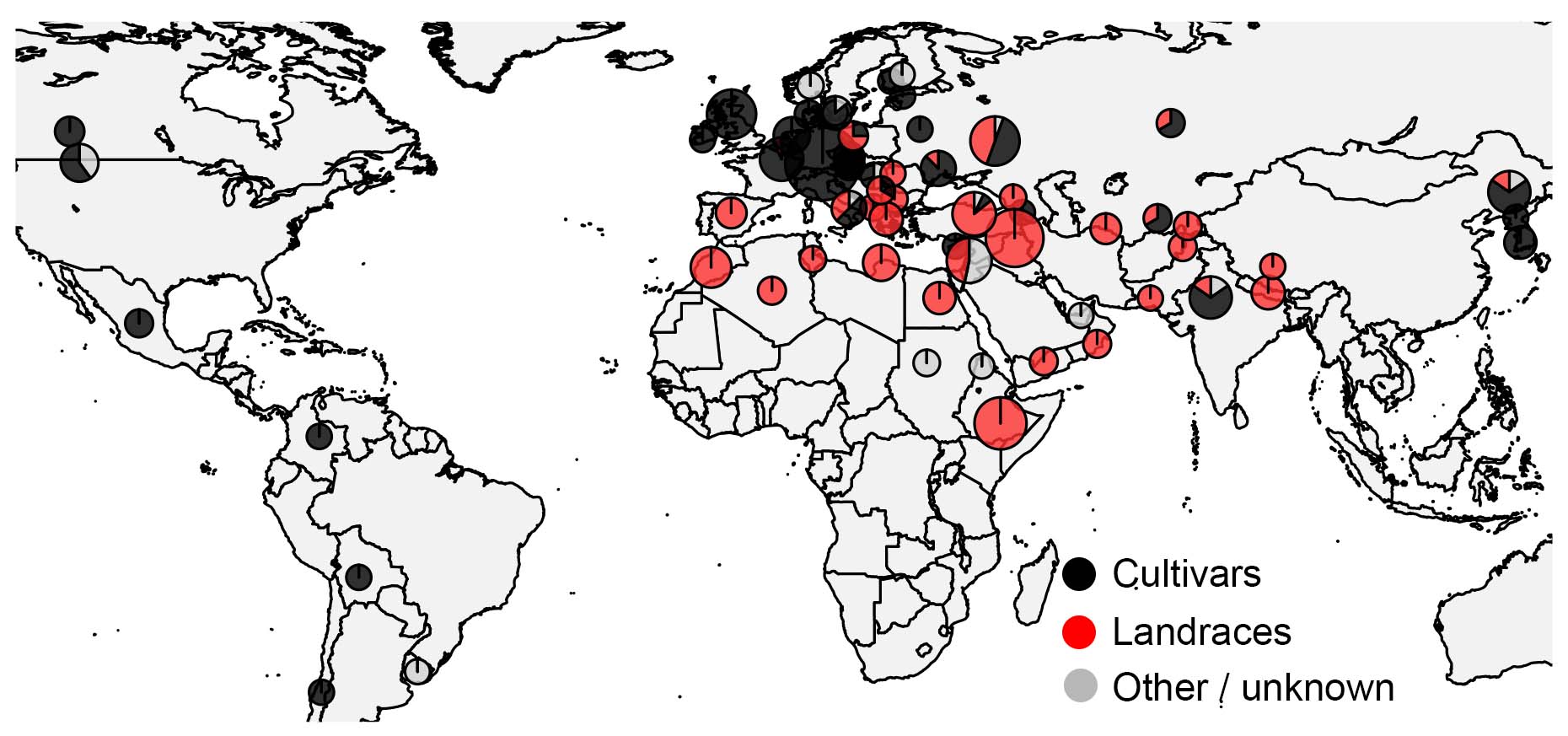

Supplement: Supplementary file 13 — Figure S13. Geographical distribution of cultivars and landraces. [file TPJ-99-1172-s013.jpg]

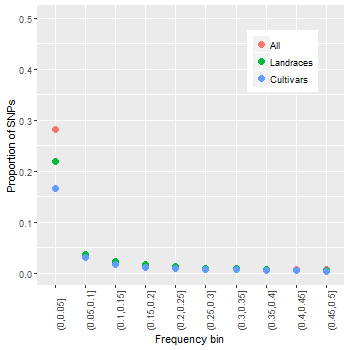

Supplement: Supplementary file 14 — Figure S14. Proportion of single nucleotide polymorphisms within minor allele frequency bins. [file TPJ-99-1172-s014.tiff]
