# Supplementary material for: Exome sequences and multi‐environment field trials elucidate the genetic basis of adaptation in barley
Source: Plant J. 2019 Jun 27;99(6):1172–91. doi: 10.1111/tpj.14414 (PMC6851764; doi:10.1111/tpj.14414)
Supplement: Supplementary file 17 — Table S2. Number of single nucleotide polymorphisms (SNPs) per chromosome, with median and mean distances between SNPs for 403 barley genotypes characterized by exome capture. Table S4. Environmental characteristics of field trials. Table S5. Generalised heritability for days to heading, plant height, 1000‐grain weight and awn length. Table S6. Variance components and standard errors for days to heading, plant height, 1000‐grain weight and awn length. Table S7. Quantitative trait loci based on haplotype states for days to heading, plant height, grain weight and awn length. Table S8. Additive effects across trial environments for a suite of circadian clock‐related genes involved in determining days to heading in the barley crop. Table S9. Haplotype states for a suite of circadian clock‐related genes involved in determining days to heading in the barley crop, with their single nucleotide polymorphism alleles and frequencies. [file TPJ-99-1172-s017.docx]

**Table S1** Passport and phenotypic data for barley lines from the WHEALBI collection.

(See Excel file)

**Table S2** Number of SNPs per chromosome, with median and mean distances between SNPs for 403 barley genotypes characterized by exome capture. The numbers of SNPs after filtering out those with a minor allele frequency (MAF) < 0.05 for the 371 domesticated accessions used for our current analysis are also shown.

| **Chromosome** | **Number of SNPs** | **Median distance** | **Mean distance** | **Number of SNPs** |
| --- | --- | --- | --- | --- |
|  | **No MAF filter, 403 genotypes** | **(bp)** | **(bp)** | **Filter MAF ≥ 0.05, 371 genotypes** |
| 1H | 263,255 | 15 | 2,121 | 48,928 |
| 2H | 334,501 | 15 | 2,296 | 72,342 |
| 3H | 320,625 | 15 | 2,182 | 68,565 |
| 4H | 256,836 | 17 | 2,519 | 45,980 |
| 5H | 312,840 | 16 | 2,140 | 71,968 |
| 6H | 262,014 | 15 | 2,226 | 55,448 |
| 7H | 340,617 | 14 | 1,929 | 72,200 |
| Total | 2,090,688 |  |  | 435,431 |

**Table S3** Kinship matrix for 371 barley genotypes used for the genetic analysis.

(See Excel file)

**Table S4** Environmental characteristics of field trials. Rainfall was calculated as the sum over the growing season, Tmean is the mean temperature during the growing season, Thermal time is the sum of degree days during the growing season, Photop. Min and Photop max. are the minimum and maximum day length during the growing season and Vern. days is the number of days with vernalizing temperatures (≥4 Celsius and ≤9 Celsius). Growing season length was defined for each environment as the period between sowing and the mean heading date, plus 40 days (a proxy for maturity)

| **Planting** | **Location** | **Planting**  **Date**  **(DD-MM-YY)** | **Location** | **Lat.** | **Long.** | **Rain**  **(mm)** | **Tmean**  **(^o^C)** | **Thermal**  **time**  **(^o^Cd)** | **Photop.**  **Min. (h)** | **Photop.**  **Max. (h)** | **Vern.**  **days** |
| --- | --- | --- | --- | --- | --- | --- | --- | --- | --- | --- | --- |
| Spring | Hungary | 11-03-15 | Hungary | 47.3 ^o^N | 18.8 ^o^E | 105 | 13.34 | 1293.55 | 11.60 | 15.60 | 25 |
|  | Scotland | 03-03-15 | Scotland | 56.5 ^o^N | 3.1 ^o^W | 239 | 11.3 | 1213.34 | 13.27 | 17.18 | 47 |
|  |  |  |  |  |  |  |  |  |  |  |  |
| Winter | Hungary | 20-10-14 | Hungary | 47.3 ^o^N | 18.8 ^o^E | 315 | 8.31 | 2150.25 | 8.39 | 15.61 | 56 |
|  | Italy | 27-10-14 | Italy | 44.9 ^o^N | 9.9 ^o^E | 651 | 8.96 | 1952.52 | 8.70 | 15.12 | 59 |
|  | Scotland | 29-10-14 | Scotland | 56.5 ^o^N | 3.1 ^o^W | 450 | 7.14 | 1741.29 | 6.82 | 17.18 | 75 |

**Table S5** Generalised heritability for days to heading, plant height, 1,000 grain weight and awn length across barley trial environments.

| **Planting** | **Location** | **DTH** | **Height** | **Grain weight** | **Awn length** |
| --- | --- | --- | --- | --- | --- |
| Spring | Hungary | 0.93 | 0.80 |  |  |
|  | Scotland | 0.98 | 0.95 | 0.94 | 0.92 |
|  |  |  |  |  |  |
| Winter | Hungary | 0.91 | 0.74 | 0.69 |  |
|  | Italy | 0.84 | 0.81 |  | 0.93 |
|  | Scotland | 0.87 | 0.74 | 0.90 | 0.83 |

**Table S6** Variance components and standard errors for days to heading, plant height, 1,000 grain weight and awn length across barley trial environments.

| **Component** | **Days to heading** | | **Plant height** | | **Grain weight** | | **Awn length** | |
| --- | --- | --- | --- | --- | --- | --- | --- | --- |
|  | **Variance** | **s.e.** | **Variance** | **s.e.** | **Variance** | **s.e.** | **Variance** | **s.e.** |
| Geno | 25.93 | 2.10 | 111.47 | 8.91 | 34.98 | 2.94 | 2.14 | 0.20 |
| Geno.Environment | 15.35 | 0.51 | 56.19 | 1.87 | 22.64 | 0.86 | 1.74 | 0.08 |
| Number of environments | 5 | | 5 | | 4 | | 3 | |

**Table S7** QTLs based on haplotype states, above a threshold of -log_10_(p)=4.0, for days to heading, plant height, grain weight and awn length for 371 domesticated barley lines based on multi-environment genome-wide association scans. Chromosome positions, number of haplotype states and –log_10_(p) values are shown.

| **Trait** | **Haplotype block** | **Chromosome** | **Gene start (Mbp)** | **Gene end (Mbp)** | **Haplotype states** | **-log_10_(p)** |
| --- | --- | --- | --- | --- | --- | --- |
| Days to heading | HORVU1Hr1G019500 block1 | 1H | 73,807,417 | 73,810,905 | 5 | 5.24 |
|  | HORVU1Hr1G049420 block1 | 1H | 365,874,545 | 365,875,282 | 4 | 6.55 |
|  | HORVU1Hr1G066340 block1 | 1H | 471,968,224 | 471,970,801 | 3 | 4.95 |
|  | HORVU2Hr1G013450 block1 | 2H | 29,201,388 | 29,204,611 | 5 | 5.24 |
|  | HORVU2Hr1G072060 block1 | 2H | 516,123,256 | 516,124,687 | 5 | 10.10 |
|  | HORVU2Hr1G086920 block1 | 2H | 625,383,512 | 625,387,717 | 3 | 6.40 |
|  | HORVU2Hr1G113880 block1 | 2H | 730,027,508 | 730,030,208 | 6 | 4.98 |
|  | HORVU3Hr1G010530 block1 | 3H | 23,184,058 | 23,187,544 | 4 | 11.45 |
|  | HORVU3Hr1G095520 block1 | 3H | 650,275,916 | 650,278,652 | 3 | 9.52 |
|  | HORVU4Hr1G052170 block1 | 4H | 431,436,720 | 431,448,817 | 2 | 4.85 |
|  | HORVU4Hr1G072060 block1 | 4H | 583,307,616 | 583,310,462 | 3 | 4.34 |
|  | HORVU4Hr1G088390 block3 | 4H | 640,491,514 | 640,497,006 | 8 | 4.51 |
|  | HORVU5Hr1G025900 block1 | 5H | 144,293,793 | 144,295,833 | 3 | 4.26 |
|  | HORVU5Hr1G074750 block1 | 5H | 544,823,015 | 544,824,869 | 4 | 6.08 |
|  | HORVU5Hr1G114250 block2 | 5H | 643,433,504 | 643,435,944 | 8 | 4.13 |
|  | HORVU7Hr1G024570 block1 | 7H | 39,476,773 | 39,487,000 | 6 | 5.10 |
|  |  |  |  |  |  |  |
| Plant height | HORVU1Hr1G039720 block1 | 1H | 279,477,858 | 279,483,318 | 3 | 4.43 |
|  | HORVU1Hr1G063420 block1 | 1H | 455,258,946 | 455,262,203 | 7 | 4.11 |
|  | HORVU2Hr1G013450 block2 | 2H | 29,201,388 | 29,204,611 | 5 | 6.40 |
|  | HORVU2Hr1G111930 block1 | 2H | 724,470,419 | 724,474,002 | 6 | 4.95 |
|  | HORVU3Hr1G090980 block2 | 3H | 634,078,038 | 634,081,600 | 3 | 5.99 |
|  | HORVU4Hr1G037290 block1 | 4H | 278,359,151 | 278,363,795 | 3 | 4.31 |
|  | HORVU4Hr1G075360 block2 | 4H | 598,065,181 | 598,068,656 | 4 | 5.34 |
|  | HORVU6Hr1G067980 block1 | 6H | 471,399,065 | 471,399,961 | 2 | 4.52 |
|  | HORVU7Hr1G024990 block1 | 7H | 41,146,333 | 41,147,786 | 3 | 5.58 |
|  | HORVU7Hr1G111940 block1 | 7H | 634,827,020 | 634,828,272 | 4 | 4.42 |
|  |  |  |  |  |  |  |
| Grain weight | HORVU1Hr1G076650 block1 | 1H | 515,119,929 | 515,123,865 | 6 | 4.87 |
|  | HORVU2Hr1G074420 block1 | 2H | 537,376,624 | 537,377,559 | 3 | 5.45 |
|  | HORVU2Hr1G091720 block1 | 2H | 649,447,408 | 649,449,948 | 4 | 7.23 |
|  | HORVU3Hr1G010540 block1 | 3H | 23,187,997 | 23,189,987 | 5 | 4.16 |
|  | HORVU3Hr1G095620 block1 | 3H | 650,539,124 | 650,540,282 | 3 | 5.71 |
|  | HORVU4Hr1G007020 block1 | 4H | 17,438,279 | 17,446,158 | 3 | 15.69 |
|  | HORVU7Hr1G110570 block1 | 7H | 630,786,598 | 630,787,627 | 3 | 4.17 |
|  |  |  |  |  |  |  |
| Awn length | HORVU1Hr1G090780 block1 | 1H | 547,045,000 | 547,052,114 | 5 | 6.63 |
|  | HORVU2Hr1G094630 block1 | 2H | 665,621,578 | 665,623,533 | 4 | 4.00 |
|  | HORVU7Hr1G010340 block2 | 7H | 14,579,369 | 14,584,070 | 4 | 4.58 |
|  | HORVU7Hr1G045470 block1 | 7H | 144,191,326 | 144,283,714 | 7 | 4.26 |

**Table S8** Additive effects across trial environments for a suite of circadian clock-related genes involved in determining days to heading (DTH) in the barley crop. Effects are shown as change in DTH compared to the most common haplotype state, *a*. For genes *HvPPD-H1* and *HvCEN*, haplotype states related to known causal mutations as presented in the literature (Turner *et al*., 2005 and Comadran *et al*., 2012, respectively) are shown. For each gene, haplotype states are ordered and labelled by frequency of occurrence, which varies greatly. Effects are therefore more definitive for the more common haplotype states closer to the top of the table for each gene.

| **Chromo** | **Gene** | **Horvu** | **Haplotype state** | **n** | **Hungary spring** | **Scotland spring** | **Hungary winter** | **Italy winter** | **Scotland winter** |
| --- | --- | --- | --- | --- | --- | --- | --- | --- | --- |
| 1H | *HvFT3* | HORVU1Hr1G076430 | *a* | 187 | 0.0 | 0.0 | 0.0 | 0.0 | 0.0 |
|  |  |  | *b* | 123 | 0.0 | 1.4 | 1.8 | 1.3 | -0.2 |
|  |  |  | *c* | 61 | -1.3 | -1.7 | -0.1 | -0.2 | -2.3 |
|  | *HvELF3* | HORVU1Hr1G094980 | *a* | 119 | 0.0 | 0.0 | 0.0 | 0.0 | 0.0 |
|  |  |  | *b* | 118 | 2.1 | 1.8 | 0.6 | 2.8 | 1.6 |
|  |  |  | *c* | 54 | 1.5 | 1.4 | 0.8 | 1.4 | 2.5 |
|  |  |  | *d* | 27 | 1.9 | 0.4 | 1.9 | 2.8 | 2.0 |
|  |  |  | *e* | 22 | -3.9 | -3.2 | -4.1 | -4.6 | -3.0 |
|  |  |  | *f* | 12 | 3.2 | -2.0 | -0.8 | -1.8 | -1.9 |
|  |  |  | *g* | 10 | -1.7 | -0.6 | -2.1 | -3.8 | -4.1 |
|  |  |  | *h* | 9 | -1.4 | -2.9 | -0.7 | -2.5 | -2.3 |
| 2H | *HvPPD-H1* | HORVU2Hr1G013400 | *a* (insensitive) | 110 | 0.0 | 0.0 | 0.0 | 0.0 | 0.0 |
|  |  |  | *b* (insensitive) | 62 | -1.6 | -0.8 | -0.5 | 0.0 | 0.4 |
|  |  |  | *c* (sensitive) | 39 | -2.0 | -2.5 | 2.6 | 0.9 | 2.2 |
|  |  |  | *d* (sensitive) | 38 | -2.6 | -4.7 | 2.4 | 1.6 | 1.6 |
|  |  |  | *e* (sensitive) | 34 | -2.1 | -1.3 | 2.2 | 0.8 | 2.5 |
|  |  |  | *f* (sensitive) | 31 | -1.2 | -4.3 | 4.2 | 3.5 | 1.6 |
|  |  |  | *g* (sensitive) | 30 | -5.4 | -5.2 | -1.3 | -5.3 | -1.8 |
|  |  |  | *h* (sensitive) | 27 | -1.4 | -0.6 | 3.9 | 3.4 | 1.2 |
|  | *HvCEN* | HORVU2Hr1G072750 | *a* (late) | 197 | 0.0 | 0.0 | 0.0 | 0.0 | 0.0 |
|  |  |  | *b* (early) | 119 | -2.7 | -2.8 | -5.2 | -6.5 | -6.6 |
|  |  |  | *c* (late) | 33 | 1.5 | 2.3 | -2.4 | 1.1 | -2.6 |
|  |  |  | *d* (early) | 22 | 1.8 | -0.9 | -0.1 | -1.2 | -0.5 |
| 3H | *HvFT2* | HORVU3Hr1G027590 | *a* | 320 | 0.0 | 0.0 | 0.0 | 0.0 | 0.0 |
|  |  |  | *b* | 32 | 2.2 | 2.2 | 0.1 | 0.5 | 0.7 |
|  |  |  | *c* | 19 | 0.5 | 1.3 | 2.4 | 2.6 | 3.3 |
| 5H | *HvGRP7a* | HORVU5Hr1G002150 | *a* | 142 | 0.0 | 0.0 | 0.0 | 0.0 | 0.0 |
|  |  |  | *b* | 125 | -0.6 | -1.2 | -0.4 | -0.9 | -0.7 |
|  |  |  | *c* | 51 | -2.5 | -2.0 | -1.6 | -4.2 | -1.2 |
|  |  |  | *d* | 42 | -1.8 | -0.5 | -0.6 | -3.0 | -1.0 |
|  |  |  | *e* | 11 | -0.7 | 0.2 | -1.7 | -2.9 | -2.7 |
|  | *HvGRP7b* | HORVU5Hr1G002120 | *a* | 135 | 0.0 | 0.0 | 0.0 | 0.0 | 0.0 |
|  |  |  | *b* | 126 | -0.9 | -1.3 | -0.2 | -0.5 | -0.7 |
|  |  |  | *c* | 59 | -1.1 | -0.3 | -1.3 | -2.0 | -2.0 |
|  |  |  | *d* | 25 | -2.1 | -1.7 | -1.1 | -4.5 | -0.6 |
|  |  |  | *e* | 19 | -1.6 | -3.2 | -2.4 | -8.9 | -2.3 |
|  | *HvPRR95* | HORVU5Hr1G081620 | *a* | 189 | 0.0 | 0.0 | 0.0 | 0.0 | 0.0 |
|  |  |  | *b* | 55 | -1.3 | 0.0 | -0.5 | -1.5 | -1.8 |
|  |  |  | *c* | 47 | 0.4 | 0.7 | 0.2 | -0.2 | 0.1 |
|  |  |  | *d* | 36 | 0.3 | 0.0 | 1.6 | 0.2 | 2.0 |
|  |  |  | *e* | 22 | -1.5 | 0.1 | -0.5 | -3.4 | -2.4 |
|  |  |  | *f* | 16 | -0.6 | 1.0 | 0.0 | -0.2 | -1.1 |
|  |  |  | *g* | 6 | -2.2 | -1.7 | -1.4 | -2.9 | -4.3 |
| 6H | *HvZTLb* | HORVU6Hr1G022330 | *a* | 200 | 0.0 | 0.0 | 0.0 | 0.0 | 0.0 |
|  |  |  | *b* | 122 | -3.2 | -2.1 | -1.6 | -3.7 | -0.8 |
|  |  |  | *c* | 49 | -0.1 | 0.9 | -2.3 | -5.4 | -0.8 |
|  | *HvTOC1* | HORVU6Hr1G057630 | *a* | 78 | 0.0 | 0.0 | 0.0 | 0.0 | 0.0 |
|  |  |  | *b* | 70 | -0.8 | -0.4 | -0.3 | 0.3 | 0.2 |
|  |  |  | *c* | 65 | -0.9 | 0.6 | -0.1 | 1.5 | -0.7 |
|  |  |  | *d* | 51 | -2.5 | -0.9 | -1.6 | -1.1 | -0.3 |
|  |  |  | *e* | 30 | 1.1 | 2.7 | 1.7 | 2.8 | 2.5 |
|  |  |  | *f* | 29 | 3.6 | 6.0 | 1.5 | 1.8 | 2.2 |
|  |  |  | *g* | 14 | -2.5 | -0.6 | -0.3 | 0.7 | -0.4 |
|  |  |  | *h* | 12 | -1.1 | -0.6 | 0.4 | 2.7 | 0.1 |
|  |  |  | *i* | 12 | 0.2 | 0.7 | -0.1 | -0.4 | -0.2 |
|  |  |  | *j* | 10 | -0.5 | 1.1 | -0.5 | 0.7 | 0.4 |
|  | *HvCO2* | HORVU6Hr1G072630 | *a* | 205 | 0.0 | 0.0 | 0.0 | 0.0 | 0.0 |
|  |  |  | *b* | 48 | 0.5 | 0.8 | 1.9 | 1.1 | 0.6 |
|  |  |  | *c* | 46 | 2.1 | 2.1 | 0.0 | 0.7 | 0.9 |
|  |  |  | *d* | 32 | -0.9 | -1.0 | -1.0 | -0.4 | -0.4 |
|  |  |  | *e* | 24 | 1.2 | 1.9 | 3.1 | 5.2 | 4.3 |
|  |  |  | *f* | 16 | 2.8 | 3.4 | 1.4 | 5.0 | 4.8 |
| 7H | *HvZTLa* | HORVU7Hr1G099010 | *a* | 142 | 0.0 | 0.0 | 0.0 | 0.0 | 0.0 |
|  |  |  | *b* | 118 | -2.0 | -1.5 | -1.6 | -2.2 | -2.1 |
|  |  |  | *c* | 102 | -0.5 | 0.1 | -0.3 | -1.5 | 0.5 |
|  |  |  | *d* | 9 | -2.2 | -1.4 | 0.8 | -0.4 | 4.0 |
|  | *HvLHY* | HORVU7Hr1G070870 | *a* | 338 | 0.0 | 0.0 | 0.0 | 0.0 | 0.0 |
|  |  |  | *b* | 27 | 3.3 | 8.5 | 4.0 | 7.1 | 4.2 |
|  |  |  | *c* | 6 | -4.3 | -2.5 | -1.9 | -1.9 | -0.8 |

**Table S9** Haplotype states for a suite of circadian clock-related genes involved in determining days to heading (DTH) in the barley crop, with their SNP alleles and frequencies. For SNP alleles, 0 = most common SNP allele (reference) and 2 = the least common SNP allele.

| **Chromo** | **Gene** | **Horvu** | **Haplotype state** | SNP alleles |
| --- | --- | --- | --- | --- |
| 1H | *HvFT3* | HORVU1Hr1G076430 | *a* | 00 |
|  |  |  | *b* | 02 |
|  |  |  | *c* | 22 |
|  | *HvELF3* | HORVU1Hr1G094980 | *a* | 00200020000000002200002 |
|  |  |  | *b* | 02000002222002200002020 |
|  |  |  | *c* | 00020000000020000020200 |
|  |  |  | *d* | 20002220222202020002020 |
|  |  |  | *e* | 00000000000000002000000 |
|  |  |  | *f* | 00000000000000000002000 |
|  |  |  | *g* | 00000000000000002000002 |
|  |  |  | *h* | 00002220222002000002020 |
| 2H | *HvPPD-H1* | HORVU2Hr1G012400 | *a* (insensitive) | 2020200002020200020000202000200000200200000200 |
|  |  |  | *b* (insensitive) | 2020200002020200020000202000000000200200000200 |
|  |  |  | *c* (sensitive) | 0002020000002000200200000200020020000002000020 |
|  |  |  | *d* (sensitive) | 0000000020200000000000000000000200022000022000 |
|  |  |  | *e* (sensitive) | 0002020000002000200200000200000020000002000020 |
|  |  |  | *f* (sensitive) | 0000000220000000002200000000000020000000002002 |
|  |  |  | *g* (sensitive) | 0000000020200000000000000000000000020000002000 |
|  |  |  | *h* (sensitive) | 0200002020000022000022020222002002000022200000 |
|  | *HvCEN* | HORVU2Hr1G072750 | *a* (late) | 000 |
|  |  |  | *b* (early) | 002 |
|  |  |  | *c* (late) | 200 |
|  |  |  | *d* (early) | 022 |
| 2H | *HvFT2* | HORVU2Hr1G027590 | *a* | 000000 |
|  |  |  | *b* | 220002 |
|  |  |  | *c* | 022220 |
| 5H | *HvGRP7a* | HORVU5Hr1G002150 | *a* | 022200002 |
|  |  |  | *b* | 200000020 |
|  |  |  | *c* | 000000200 |
|  |  |  | *d* | 000022000 |
|  |  |  | *e* | 000000000 |
|  | *HvGRP7b* | HORVU5Hr1G002120 | *a* | 022200002 |
|  |  |  | *b* | 200000020 |
|  |  |  | *c* | 000000200 |
|  |  |  | *d* | 000022000 |
|  |  |  | *e* | 000000000 |
|  | *HvPRR95* | HORVU5Hr1G081620 | *a* | 2002000000000000 |
|  |  |  | *b* | 0000000020022200 |
|  |  |  | *c* | 0000200200000000 |
|  |  |  | *d* | 0220022002202002 |
|  |  |  | *e* | 0000000200000020 |
|  |  |  | *f* | 0000200200000020 |
|  |  |  | *g* | 0220022000002002 |
| 6H | *HvZTLb* | HORVU6Hr1G022220 | *a* | 000 |
|  |  |  | *b* | 220 |
|  |  |  | *c* | 002 |
|  | *HvTOC1* | HORVU6Hr1G057620 | *a* | 000000202002000 |
|  |  |  | *b* | 220222020200000 |
|  |  |  | *c* | 220222020200020 |
|  |  |  | *d* | 000000002002202 |
|  |  |  | *e* | 002000200000000 |
|  |  |  | *f* | 000000200022000 |
|  |  |  | *g* | 000000202000000 |
|  |  |  | *h* | 000000200000000 |
|  |  |  | *i* | 000002200200000 |
|  |  |  | *j* | 000000002002002 |
|  | *HvCO2* | HORVU6Hr1G072620 | *a* | 000000000000000000000000000 |
|  |  |  | *b* | 220002200002220020000020020 |
|  |  |  | *c* | 222222020000200020222002222 |
|  |  |  | *d* | 000000002020000022000200020 |
|  |  |  | *e* | 000000000200002200000000020 |
|  |  |  | *f* | 020000000000200020000000020 |
| 7H | *HvZTLa* | HORVU7Hr1G099010 | *a* | 0022220 |
|  |  |  | *b* | 2000002 |
|  |  |  | *c* | 0200000 |
|  |  |  | *d* | 0002000 |
|  | *HvLHY* | HORVU7Hr1G070870 | *a* | 000000 |
|  |  |  | *b* | 222222 |
|  |  |  | *c* | 020000 |
