# Supplementary material for: Exome sequences and multi‐environment field trials elucidate the genetic basis of adaptation in barley
Source: Plant J. 2019 Jun 27;99(6):1172–91. doi: 10.1111/tpj.14414 (PMC6851764; doi:10.1111/tpj.14414)
Supplement: Supplementary file 18 [file TPJ-99-1172-s018.docx]

**Fig. S1** SNP density expressed as the number of SNPs per Mbp of sequence for barley chromosomes for 403 barley genotypes.

**Fig. S2** Relationship among genetic and geographic features for a subset of 174 spring habit domesticated barley genotypes. (a) Eigenvalues resulting from a spatial principal component analysis (sPCA). Positive eigenvalues (spatial autocorrelation) correspond to relatively greater genetic similarity between geographically closer individuals (larger eigenvalues indicate that genetic similarities are driven by the geographical distance between sites of origin). Conversely, negative eigenvalues correspond to greater dissimilarity between neighbours. Genetic differentiation is better explained by regional than local structuring (respective start points on a spectrum from red to blue). (b) Eigenvalues of sPCA (denoted $\lambda_{i}$ with i = 1, . . . , r, where $\lambda_{1}$ is the highest positive eigenvalue, and$\lambda_{r}$ is the highest negative eigenvalue), measuring spatial genetic differentiation (spatial autocorrelation according to Moran’s *I*). The first and second Eigenvalues explain the greatest amount of genetic variance and demonstrate high spatial autocorrelation, indicating the importance of components sPC1 and sPC2 for characterizing genetic differentiation across the geographical distribution of spring habit barley. (c) Spatial representation of sPC1 and sPC2 across the sampled area, indicating important trends in relation to geographic distances. Dots of the same colours indicate genetically more related genotypes (genotypes assigned to six categories).

**Fig. S3** Genetic differentiation (*F_st_*) between grouped genotypes along barley chromosomes. The *x*-axes represent physical distances. *F_st_* values are reported as moving medians for 100 bp bins. Genotypes grouped according to (a) row type (2-row versus 6- row, *N* = 191 and *N* = 175, respectively) and (b) breeding history (cultivar versus landrace, *N* = 174 and *N* = 166, respectively). Red vertical lines indicate subsets of known genes coincident with or close to highly differentiated regions.

**Fig. S4** Summary of phenotypic data for days to heading, plant height, 1,000 grain weight and awn length (respectively by figure panel row order) across multiple trial environments for each of six sub-populations of barley genotypes identified from exome capture data for 371 domesticated lines (cultivars and landraces combined; see sub-population composition in Fig. 1). Not all phenotypic traits were collected for all trials (represented by empty boxes). Considering DTH, profiles show that the season of sowing is an important driver of G×E, with the ranking of sub-populations changing across sowing season. Most of this G×E was driven by sub-population B, which contains most of the tested winter barley lines, heading relatively late in spring sown trials compared to winter sown trials.

**Fig. S5** GGE biplot for (a) days to heading, (b) plant height, (c) 1,000 grain weight, and (d) awn length for 371 domesticated barley lines across multiple trial environments. Symbol colour indicates the six sub-populations identified from exome capture data (see Fig. 1). Blue labels indicate trial environments. PC1 is related to the magnitude of the genotype main effect. Genotypes showing a large positive score for PC1 have a larger phenotypic value across environments, and genotypes showing a large negative score have smaller phenotypic values, compared to the population mean. PC2 is mostly related to G×E, with the angle between environment vectors related to their correlation. Environments with orthogonal vectors are driving G×E.

**Fig. S6** AMMI biplot for days to heading (DTH) for 371 domesticated barley lines across multiple trial environments. Symbol colour represents the six sub-populations identified from exome capture data (see Fig. 1). Blue labels indicate trial environments. Genotypes deviating most positively or negatively from zero contribute more to the G×E interaction (i.e., genotypes with scores close to zero deviate little from the general population response along the environmental gradient). The AMMI 1 axis indicates the contrast between spring sown and winter sown trials. Sub-population B shows a more negative average score, indicating that its DTH is relatively more delayed in spring trials than that of the other sub-populations. The AMMI 2 axis indicates the contrast between the Italy winter trial and other winter trials in Scotland and Hungary.

**Fig. S7** Manhattan plots for (a) days to heading, (b) plant height, (c) 1,000 grain weight and (d) awn length for 371 domesticated barley lines based on multi-environment genome-wide association scans of haplotype states. Vertical lines indicate positions of some important known genes reported in the literature. The *x*-axes represent physical distances.

**Fig. S8** Percentage of variance in days to heading for 371 barley genotypes explained by haplotype states for a suite of known circadian clock-related genes. (a) Genotype effect. (b) G×E effect. Assessed genes were identified by Calixto *et al*. (2015) and analysed initially at an exomic level by Russell *et al.* (2016).

**Fig. S9** Proportions of genotypes carrying each haplotype state for a suite of known circadian clock-related genes for cultivars (*N* = 174) and landraces (*N* = 166). Proportions for the balance of the 371 total domesticated barley genotypes that were uncategorised by breeding history (*N* = 31) are also shown. The number of occurrences of each haplotype state (n) across the entire sample is shown on the *x*-axis, ordered by frequency of overall occurrence (from left to right).

**Fig. S10** The proportion of genotypes that belong to the category ‘landrace’ from all genotypes carrying (a) the *most common* haplotype state and (b) the *least common* haplotype state, across all exomic haplotype blocks. For a large proportion of genotypes the rarest haplotype state occurs more often in landraces than in cultivars.

**Fig. S11** Mean days to heading (DTH) of specific *HvPPD-H1* haplotype states across five environments for 174 spring habit domesticated barley lines. Each circle represents genotypes whose site of origin is within the same latitude bin of 10 degrees. The *x*-axis was centred by the latitude of each trial site (i.e., points to the right of the *x*/*y* intercept are south of the trial site). Genotypes were classified as (a) All genotypes. (b) Cultivars. (c) Landraces. The point size indicates the relative number of accessions with a particular *HvPPD-H1* haplotype in a specific latitude bin.

**Fig. S12** Geographical distribution of haplotype states for clock-related *HvPPD-H1* and *HvCEN* genes. (a) *HvPPD-H1*. (b) *HvCEN*. In both cases, data for 371 domesticated barley genotypes are shown. Pie chart size reflects the number of accessions collected from a particular geographical region, while slice size shows the proportion of accessions with a particular haplotype state. The alphabetic order of the haplotype states coincides with their frequency ranking. Geographic partitioning of haplotype states, especially latitudinally, is most evident for *HvCEN*. The geographic partitioning of variation at these genes combined across latitude and longitude is consistent with the overall partitioning of exomic variation observed in Fig. 1a and with the pattern for a spring habit barley subset observed in Fig. S2.

**Fig. S13** Geographical distribution of 371 domesticated barley genotypes categorised by breeding history as cultivars (*N* = 174), landraces (*N* = 166) or of undefined breeding history (*N* = 31). Pie chart size reflects the number of genotypes collected from a particular geographical region, while slice size shows the proportion of genotypes belonging to a particular breeding history sub-category.

**Fig. S14** Proportion of SNPs within minor allele frequency (MAF) bins. Values are calculated across all 371 domesticated barley genotypes, for landraces only (*N* = 166) and for cultivars only (*N* = 174).

**Table S1** Passport and phenotypic data for barley lines from the WHEALBI collection.

**Table S2** Number of SNPs per chromosome, with median and mean distances between SNPs for 403 barley genotypes characterized by exome capture. The numbers of SNPs after filtering out those with a minor allele frequency (MAF) < 0.05 for the 371 domesticated accessions used for our current analysis

**Table S3** Kinship matrix for 371 barley genotypes used for the genetic analysis.

**Table S4** Environmental characteristics of field trials. Rainfall was calculated as the sum over the growing season, Tmean is the mean temperature during the growing season, Thermal time is the sum of degree days during the growing season, Photop. Min and Photop max. are the minimum and maximum day length during the growing season and Vern. days is the number of days with vernalizing temperatures (≥4 Celsius and ≤9 Celsius). Growing season length was defined for each environment as the period between sowing and the mean heading date, plus 40 days (a proxy for maturity)

**Table S5** Generalised heritability for days to heading, plant height, 1,000 grain weight and awn length across barley trial environments.

**Table S6** Variance components and standard errors for days to heading, plant height, 1,000 grain weight and awn length across barley trial environments.

**Table S7** QTLs based on haplotype states, above a threshold of -log_10_(p)=4.0, for days to heading, plant height, grain weight and awn length for 371 domesticated barley lines based on multi-environment genome-wide association scans. Chromosome positions, number of haplotype states and –log_10_(p) values are shown.

**Table S8** Additive effects across trial environments for a suite of circadian clock-related genes involved in determining days to heading (DTH) in the barley crop. Effects are shown as change in DTH compared to the most common haplotype state, *a*. For genes *HvPPD-H1* and *HvCEN*, haplotype states related to known causal mutations as presented in the literature (Turner *et al*., 2005 and Comadran *et al*., 2012, respectively) are shown. For each gene, haplotype states are ordered and labelled by frequency of occurrence, which varies greatly. Effects are therefore more definitive for the more common haplotype states closer to the top of the table for each gene.

**Table S9** Haplotype states for a suite of circadian clock-related genes involved in determining days to heading (DTH) in the barley crop, with their SNP alleles and frequencies. For SNP alleles, 0 = most common SNP allele (reference) and 2 = the least common SNP allele.
